# Conjugated Dienoic Acid Peroxides as Substrates in *Chaetopterus* Bioluminescence System

**DOI:** 10.3390/ijms24119466

**Published:** 2023-05-30

**Authors:** Renata I. Zagitova, Konstantin V. Purtov, Aleksandr S. Shcheglov, Konstantin S. Mineev, Maxim A. Dubinnyi, Ivan N. Myasnyanko, Olga A. Belozerova, Vera G. Pakhomova, Valentin N. Petushkov, Natalia S. Rodionova, Vladislav A. Lushpa, Elena B. Guglya, Sergey Kovalchuk, Valeri B. Kozhemyako, Jeremy D. Mirza, Anderson G. Oliveira, Ilia V. Yampolsky, Zinaida M. Kaskova, Aleksandra S. Tsarkova

**Affiliations:** 1 Shemyakin-Ovchinnikov Institute of Bioorganic Chemistry, Russian Academy of Sciences, Miklukho-Maklaya, 16/10, 117997 Moscow, Russiaivan.n.myasnyanko@gmail.com (I.N.M.); o.belozyorova@gmail.com (O.A.B.); lushpa@phystech.edu (V.A.L.); ivyamp@gmail.com (I.V.Y.); zkaskova@ibch.ru (Z.M.K.); 2 Institute of Biophysics SB RAS, Federal Research Center “Krasnoyarsk Science Center SB RAS”, Akademgorodok 50/50, 660036 Krasnoyarsk, Russia; purtovk@ibp.ru (K.V.P.); valnat@yandex.ru (V.N.P.);; 3 Institute of Translational Medicine, Pirogov Russian National Research Medical University, Ostrovityanova 1, 117997 Moscow, Russia; 4 Department of Biological and Medical Physics, Moscow Institute of Physics and Technology (State University), 9 Institutskiy per., Dolgoprudny, 141700 Moscow, Russia; 5 Federal Research Center “Krasnoyarsk Science Center SB RAS”, Akademgorodok 50, 660036 Krasnoyarsk, Russia; vgpakhomova@mail.ru; 6 Central Research Laboratory, Pacific State Medical University, Ostryakova 2, 690002 Vladivostok, Russia; 7 School of Biosciences, University of Birmingham, Edgbaston, Birmingham B15 2TT, UK; jeremymirza.unifesp@gmail.com; 8 Department of Chemistry and Biochemistry, Yeshiva University, 245 Lexington Ave, New York, NY 10016, USA; a.oliveira@yu.edu

**Keywords:** ferroptosis, lipid peroxidation, bioluminescence, luciferase, luciferin, bioimaging, biomedical research, parchment worm, *Chaetopterus*

## Abstract

Biochemistry of bioluminescence of the marine parchment tubeworm *Chaetopterus* has been in research focus for over a century; however, the results obtained by various groups contradict each other. Here, we report the isolation and structural elucidation of three compounds from *Chaetomorpha linum* algae, which demonstrate bioluminescence activity with *Chaetopterus* luciferase in the presence of Fe^2+^ ions. These compounds are derivatives of polyunsaturated fatty acid peroxides. We have also obtained their structural analogues and demonstrated their activity in the bioluminescence reaction, thus confirming the broad substrate specificity of the luciferase.

## 1. Introduction

Bioluminescence, or emission of light by living organisms, is widespread in nature. The process is normally caused by the oxidation of a special substrate, named luciferin, by oxygen from the air, catalyzed by an enzyme, luciferase [1]. Bioluminescence systems demonstrate amazing diversity: more than 40 different bioluminescent mechanisms are known; however, structures for only 10 luciferins are elucidated [2]. Bioluminescence is widely applied in biotechnology [3]. Investigation of the least-studied bioluminescence systems is of both fundamental and practical interest [4] ensuring the development of new methods in bioimaging, drug screening, environmental monitoring, and other applications.

The glowing of the marine polychaete worm *Chaetopterus* was first described at the end of the 19th century, but its mechanism is still unknown [5]. Interestingly, *Chaetopterus* emits light in two ways: through secreted luminous mucus and by short “flashes” of posterior segments of the body [6]. The first attempt to study *Chaetopterus* bioluminescence was made by Shimomura and Johnson, who used the 12th segment of the body of the worm, which contains glands that secrete luminous mucus [7]. They showed that the *Chaetopterus* bioluminescence system consists of five components: a 128–130 kDa dimeric protein (which was assumed as a photoprotein), two cofactors (presumably a nucleoprotein and a lipid), hydrogen peroxide, and ferrous ions. At that time, the structures of the molecular substrates remained unknown [6]. Modern studies of the luminous mucus secreted by living worms showed different results: hydrogen peroxide inhibited the bioluminescence reaction, while the addition of Fe^2+^ ions enhanced it by only 1.2–3 times [8]. Our recent studies showed that the *Chaetopterus* bioluminescence system consists of an enzyme (luciferase) and a low-molecular-weight substrate (luciferin) and requires a cofactor—ferrous ions—for luminescence [9]. In this paper, we discuss the identification, synthesis, and study of the bioluminescent activity of *Chaetopterus* luciferase substrates.

## 2. Results and Discussion

### 2.1. Extraction, Purification, and Structural Elucidation of Bioluminescent Compounds from Chaetomorpha Linum Biomass

Previously, we reported the characterization of the *Chaetopterus variopedatus* bioluminescence system components, including luciferin–luciferase activity upon the addition of exogenous ferrous ions Fe^2+^, as well as the preparation of partially purified *C. variopedatus* luciferase [9]. However, despite the fact that the initial extracts obtained from *C. variopedatus* biomass demonstrated high bioluminescent activity, the isolation of active luciferin in sufficient amounts was not possible. Heating led to a complete loss of luciferin luminescent activity; therefore, we could not obtain a “hot” extract. Solvent extracts from biomass and primary aqueous extracts also did not demonstrate significant luminescent activity of luciferin. The most efficient extract of a primary biomass homogenate would have required processing several tens of kilograms of *C. variopedatus* biomass to allow luciferin in amounts sufficient for LC–MS (liquid chromatography–mass-spectrometry) analysis, which was unrealistic to accomplish.

Inspired by a previous strategy used for the elucidation of fungal luciferin [10], we decided to look for precursors and analogues of *C. variopedatus* luciferin in other marine organisms. Substances exhibiting luciferin activity with *C. variopedatus* luciferase were found in a number of organisms of both plant and animal origin (soft corals, cephalopods, algae), with seaweed *C. linum* containing the highest amount of it. Hence, we focused on the development of a method for the isolation, purification, and elucidation of the chemical structure of active bioluminescent compounds from algae biomass.

**Figure 1:**
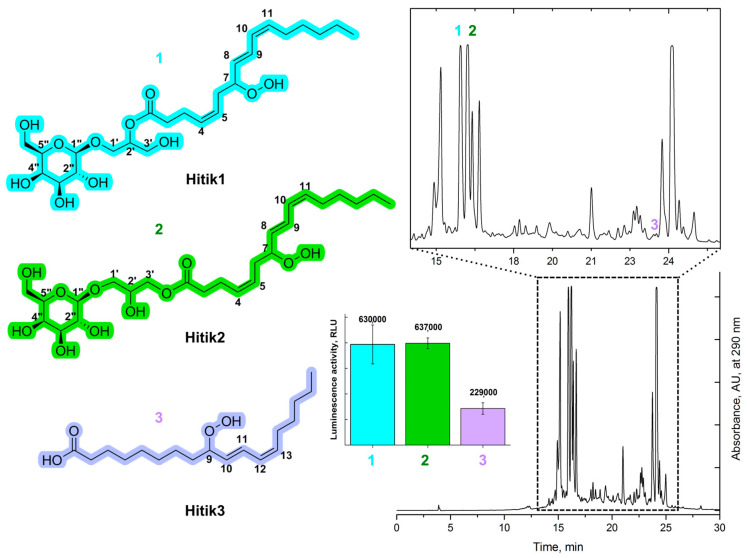
Bioluminescent compounds isolated from ethanol extract of the algae *Chaetomorpha linum*. On the left—structures of the compounds **Hitik1**, **Hitik2**, **Hitik3** (with selective atom numbering) corresponding to the chromatographic peaks 1, 2, and 3 (indicated on the inset). On the right—chromatographic profile of an ethanol extract sample on a ZORBAX Eclipse XDB-C18 column (9.4 × 250 mm). In the middle—mean luminescence activity of isolated peaks with *C. variopedatus* luciferase in RLU (relative light units) (measured in triplicate, expressed as mean ± SD (n = 3) and analyzed using Student’s *t*-test; differences were considered significant at *p* < 0.05).

Ethanol extraction from wet *C. linum* biomass followed by RP-HPLC (reversed-phase high-performance liquid chromatography) allowed us to obtain three substrates, which demonstrate bioluminescent activity with luciferase-containing fraction, in sufficient amounts for further analysis. The compounds were named **Hitik1**, **Hitik2,** and **Hitik3** according to the order in which they were eluted (Figure 1, Figure S1, ).

Structural elucidation of purified compounds started with NMR (nuclear magnetic resonance) analysis of 2D DQF-COSY (double quantum filtered correlated spectroscopy), 2D ^1^H-^13^C HSQC (heteronuclear single quantum coherence), 2D ^1^H-^13^C HMBC (heteronuclear multiple bond correlation), and 2D ^1^H-^13^C HSQC-TOCSY (total correlation spectroscopy) spectra recorded in DMSO-*d*_6_ (dimethyl sulfoxide). The high quality of the NMR spectra obtained allowed for straightforward elucidation, since all quaternary carbons were observed in HMBC and 1D ^13^C spectra, and all labile OH protons were clearly visible in DMSO solution. First, we determined the structure of **Hitik3**. According to the NMR spectra, **Hitik3** is a linear molecule and contains a carboxyl group (174.89 ppm and a broad proton signal at ~12 ppm), two double bonds, and a hydroperoxide group (singlet proton at 11.27 ppm and ternary carbon at 85.1 ppm). The structure of **Hitik3** (Figure 1) corresponds to (10*E*,12*Z*)-9-hydroperoxyoctadeca-10,12-dienoic acid or 9-(*E,Z*)-HPODE. The configuration of double bonds was determined on the basis of *J*-coupling values, which were equal to 15 Hz for the bond 10–11 and to 11 Hz for the bond 12–13. The structure of **Hitik3** is also supported by MS analysis with the mass of molecular ion M-H^-^ equal to *m/z* 311.2231 and an additional peak in the MS at *m/z* 293.2124 (−18 Da), being the reduction product with a keto group in position 9 instead of the hydroperoxide moiety (characteristic to hydroperoxides).

Compounds **Hitik1** and **Hitik2** (Figure 1) provided indistinguishable mass spectra with the major peaks at m/z 483.2596, 501.2699, and 536.3078 in positive mode. Analysis of the NMR spectra revealed that both compounds contained three identical residues with altered modes of linkages: hexopyranose carbohydrate, glycerol, and a modified polyunsaturated fatty acid. In **Hitik1**, the fatty acid is linked to the second carbon (2′) of the glycerol moiety, while for **Hitik2**, glycerol is substituted at positions 1′ and 3′. Analysis of hexopyranose allowed us to assign it unambiguously to β-galactose based on the ^13^C chemical shifts (CASPER web server (http://www.casper.organ.su.se/casper/) [11]) and *J*-couplings. H-1″-H-2″ coupling is equal to 7 Hz, which excludes the mannose configuration and suggests the β-configuration of the anomeric carbon; and a small *J*-coupling value (less than 2 Hz) is observed between the protons H-4″ and H-5″, implying the galactose configuration of the hexapyranose. Unlike **Hitik3**, the fatty acid had a length of 16 carbon atoms and contained 3 double bonds at positions 4 (*Z*), 8 (*E*), and 10 (*Z*), with the hydroperoxide group at C-7, resulting in (4*Z*,8*E*,10*Z*)-7-hydroperoxyhexadeca-4,8,10-trienoic acid. The mass of M + H^+^ peak of the compound is equal to 519.2800, which does not correspond to any of the peaks in MS; however, 501.2699 corresponds to M − H_2_O + H^+^, 483.2596 corresponds to M – 2 × H_2_O + H^+^, while 536.3078 may correspond to M + NH_4_^+^. Most likely, compounds are not stable after ionization and immediately convert into ketones instead of the hydroperoxides. Structures of the compounds and chemical shift assignments are shown in Figure 1 and Supplementary Material Tables S1–S3 (Supporting Information).

### 2.2. Synthesis of Bioluminescent Substrate Hitik3

One of the luminescent compounds, named **Hitik3**, was identified as linoleic acid hydroperoxide at position 9 (9-(*E*,*Z*)-HPODE) using NMR and HRMS (high resolution mass-spectrometry) analysis (Figure 1). To ensure the correct elucidation of the structure of this natural product and for further studies of its possible role in the bioluminescence system of *C. variopedatus*, it was necessary to synthesize it in sufficient quantities.

**Scheme 1:**
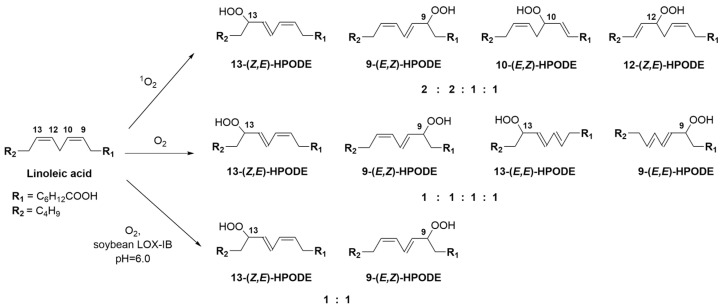
Isomers of hydroperoxyoctadecadienoic acid (HPODE) formed during oxidation of linoleic acid. Different products are formed during oxidation by singlet oxygen (^1^O_2_), atmospheric oxygen (O_2_) and soybean lipoxygenase (LOX-IB), shown with relative ratio of products [15].

There are several well-established approaches to peroxidation of polyunsaturated fatty acids (PUFA): peroxidation with lipoxygenases, singlet oxygen, or atmospheric oxygen [12,13,14]. PUFAs are usually peroxidized at different positions and with divergent configurations of double bonds in the products (Scheme 1). To obtain the target hydroperoxide 9-(*E,Z*)-HPODE, we tested two of the described approaches: oxidation by singlet oxygen, generated in the presence of methylene blue, and by soybean lipoxygenase.

**Figure 2:**
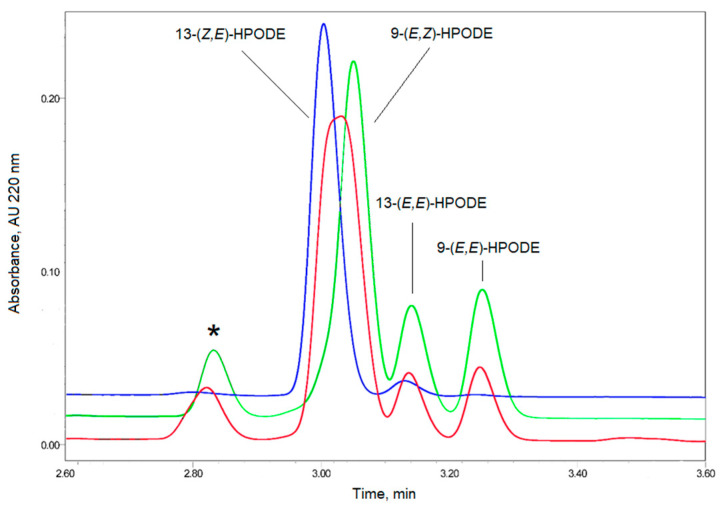
Composition of the initial mixture of linoleic acid peroxides, obtained by soybean LOX (red), HPLC-purified fractions of 9-(*E*,*Z*)-HPODE (green), and 13-(*Z*,*E*)-HPODE (blue). Represented by the overlay of analytical chromatograms (obtained on Waters Acquity BEH C18 1.7 um 2.1 × 50 mm column, detection at 220 nm). Structures of the compounds could be found on Scheme 1. *—degradation products formed from hydroperoxides.

**Figure 3:**
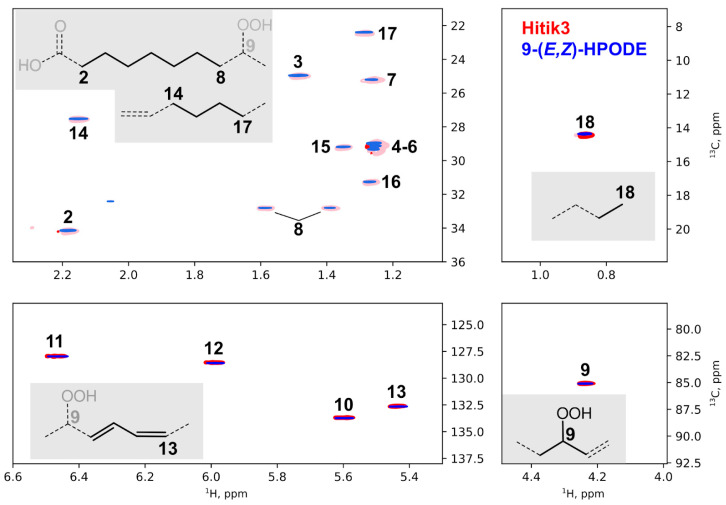
Superposition of multiplicity edited ^1^H-^13^C HSQC of Hitik3 native sample (red, 800 MHz) and synthetic 9-(*E*,*Z*)-HPODE (blue, 700 MHz). Overlay proves the identity of the compounds. Signals were unambiguously assigned (bold numbers) using ^1^H-^13^C HMBC and ^1^H-^13^C HSQC-TOCSY experiments acquired for both natural and synthetic ones. Conditions: DMSO-*d*_6_, 30 °C.

Incubation of linoleic acid with soybean lipoxygenase (LOX-IB) in 50 mM phosphate buffer at pH = 6 allowed a mixture of peroxides with 9-(*E*,*Z*)- and 13-(*Z*,*E*)-HPODE being the main components and 9-(*E*,*E*)- and 13-(*E*,*E*)-HPODE, still obtained by oxidation with oxygen,—the minor ones (Scheme 1) [16]. The preparative separation of such mixtures is a challenge due to the insignificant difference in mobility of the components and their instability, which limits the choice of possible additives in the mobile phase and forces the number of purification steps to be minimized. Normal phase chromatography allowed good separation of 9-(*E*,*Z*)- from 13-(*Z*,*E*)-HPODE; however, it was difficult to purify target 9-(*E*,*Z*)-HPODE from other two isomers (9-(*E*,*E*)- and 13-(*E*,*E*)-), so the sample of 80% purity 9-(*E*,*Z*)-HPODE was used for further experiments (Figure 2). A thorough analysis of the 2D NMR spectra obtained for the samples confirmed the structure proposed for **Hitik3** product—9-(*E*,*Z*)-HPODE (Figure 3, Supplementary Materials Figure S2, Table S4).

High amounts of PUFA peroxides are usually obtained by oxidation with lipoxygenases as catalysts. However, the application of the method is limited due to the substrate specificity of enzymes; thus, the singlet oxygen oxidation method is preferred. To study the effect of substrate structure on bioluminescence activity, we obtained peroxides of oleic (C18:1, ω-9), linoleic (C18:2, ω-6) and linolenic acids (C18:3, ω-3) by oxidation with methylene blue as a catalyst (Supplementary Materials Figure S14, Supporting Information). As the total amount of peroxide isomers obtained in the resulting mixtures is equal to the number of allyl positions, individual isolation of the compounds from the mixtures was not possible. For further activity tests, PUFA peroxides were used in the mixtures.

### 2.3. Synthesis of Hitik Structural Analogues

**Scheme 2:**
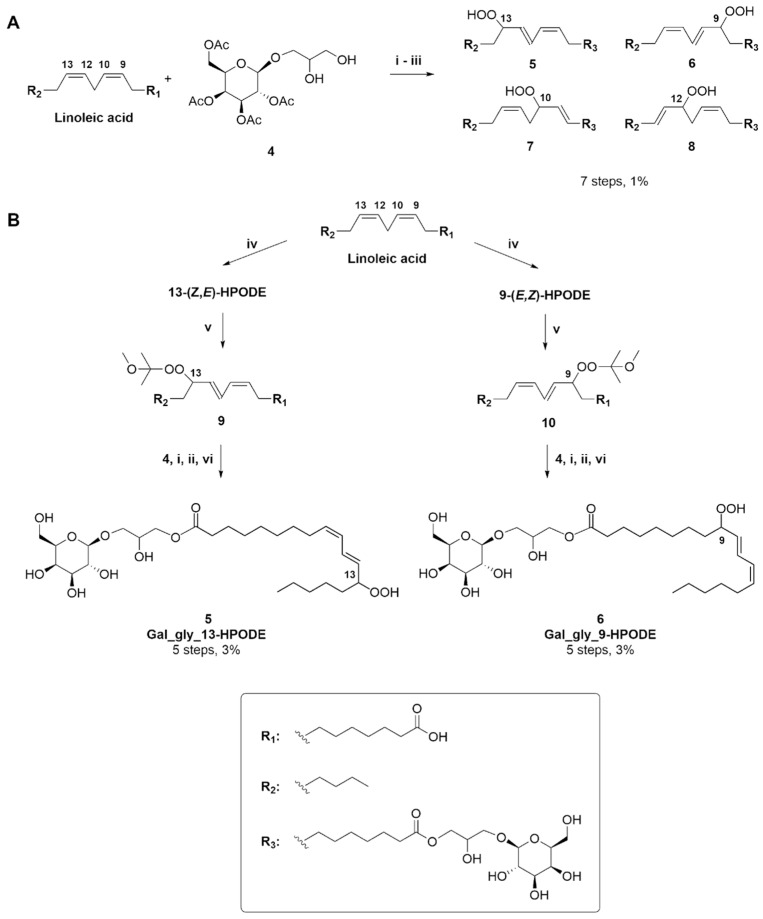
Two routes for the synthesis of β-galacto-1-linoleate-glycerol peroxides—structural analogues of **Hitik1** and **Hitik2**. (**A**). Synthesis of β-galacto-1-linoleate-glycerols with peroxidation at the last step (resulting in the mixture of products). (**B**). Preparation of individual linoleic acid peroxides, which are introduced into coupling with a galactose fragment. Reagents and conditions: (i) DCC, DMAP, CH_2_Cl_2_, r. t., overnight; (ii)—N_2_H_4_ × H_2_O, 85% EtOH, 40 °C, 4 h; (iii)—^1^O_2_, methylene blue, UV, MeOH; (iv)—O_2_, soybean LOX-IB, 50 mM phosphate buffer (pH = 6), r. t., 18h; (v)—methoxypropene, PPTs, CH_2_Cl_2_, r. t., 2 h; (vi) PPTs, CH_2_Cl_2_: MeOH (1:1), r. t., 12h. (DCC—dicyclohexylcarbodiimide, DMAP—dimetylaminopyridine, PPTs—pyridinium *p*-toluene sulfonate).

**Scheme 3:**
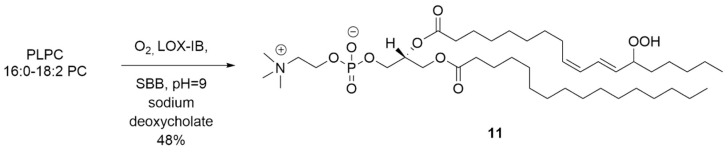
Synthesis of PLPC-OOH—1-palmitoyl-2-hydroperoxyoctadecadienoyl-*sn*-glycero-3-phosphocholine 11.

Having developed a good reproducible method for the synthesis of linoleic acid peroxides, we contemplated the possibility of using it for the synthesis of **Hitik1** and **Hitik2** compounds. These natural products contain a peroxide moiety of 4,7,10-hexadecatrienoic acid, which is much less available than linoleic acid and also does not allow the selective introduction of the OOH group. *Chaetopterus* luciferase, in its turn, appears to be a promiscuous enzyme with greater substrate specificity. For these reasons, we decided to obtain synthetic analogues of **Hitik1** and **Hitik2** substrates, containing peroxide moieties of linoleic acid (Scheme 2) and a peroxide from commercially available phospholipid 1-palmitoyl-2-linoleoyl-sn-glycero-3-phosphocholine (PLPC) (Scheme 3).

The target galactolipid peroxides **5**–**8** were synthesized according to known procedures [17,18,19,20,21] using two different synthetic routes (Scheme 2). First one included the synthesis of a conjugate of linoleic acid with galactose moiety **4** followed by peroxidation with ^1^O_2_ in the presence of methylene blue to provide a range of products **5**–**8** (Scheme 2A). The resulting mixture of peroxides was separated from the side products using normal phase chromatography, but individual compounds could not be obtained. Second pathway was less convergent and implied selective introduction of the peroxide group with lipoxygenase, followed by the protection of hydroperoxide by α-methoxyisopropyl protecting group, conjugation with a carbohydrate moiety **4** and deprotection to allow two target products **5** and **6**. PLPC peroxide **11** was obtained using the described procedure [22] (Scheme 3).

### 2.4. Bioluminescent Activity of the Synthesized Substrates

**Figure 4:**
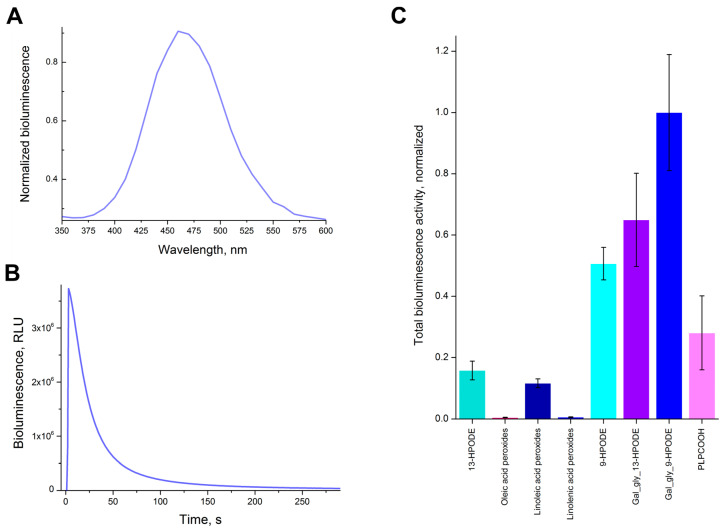
Spectrum, kinetics and activity of bioluminescence reaction of substrates with partially purified *Chaetopterus variopedatus* luciferase and Fe^2+^ ions. (**A**). Bioluminescence spectrum for 13-HPODE. (**B**). Kinetics of bioluminescence reaction of 13-HPODE. (**C**). Normalized bioluminescent activity of synthetic substrates. Activity data expressed as the net area under the curve of the luminescence reaction kinetics measured over 3 min (total bioluminescence), normalized by the total bioluminescence of Gal_gly_9-HPODE. Data are measured in triplicate, expressed as mean ± SD (n = 3), and analyzed using Student’s t-test; differences were considered significant at *p* < 0.05. 9-HPODE and 13-HPODE are 9-(*E*,*Z*)-HPODE and 13-(*Z*,*E*)-HPODE, respectively (see Scheme 1), Gal_gly_13-HPODE—compound **5**, Gal_gly_9-HPODE—compound **6** (Scheme 2), PLPCOOH—compound **11** (Scheme 3). Equipment for (**A**)—Shimadzu-UV-Mini-1240-UV-VIS Spectrophotometer (Shimadzu, Kyoto, Japan), (**B**,**C**)—custom made luminometer BLM (Oberon-K, Krasnoyarsk, Russia).

We proceeded to carry out a series of experiments to investigate the bioluminescent activity of the synthesized substrates and mixtures. All experiments were measured while mixing the substrates with partially purified *C. variopedatus* luciferase and Fe^2+^ ions (Figure 4, Figure S13, Supporting information). The maximum wavelength of bioluminescence emission for 13-HPODE is 460 nm (Figure 4A), which coincides with the data obtained for *Chaetopterus* mucus: 463 nm [23] or 455 nm [8]—indirectly supporting the hypothesis that the natural *Chaetopterus* luciferin could have the same active moiety as the **Hitik** compounds. Bioluminescence kinetics of 13-HPODE demonstrates a characteristic flash profile (Figure 4B).

The peroxide mixtures from oleic and linolenic acids were almost inactive in the bioluminescence reaction (Figure 4C, Supplementary Materials Figure S14, Supporting Information). Mixtures of linoleic acid peroxides were less active than 13-HPODE, which is probably due to the presence of 10- and 12-HPODE in the sample. The highest relative total luminescence activity was observed for the substrates, bearing a carbohydrate moiety **5** and **6** (Figure 4C), which could be explained by their better solubility in aqueous buffers. We also found that compounds with the OOH group at C-9 are more active than their C-13 isomers (Figure S15, Supporting Information). Interestingly, PLPC peroxide (PLPCOOH) demonstrated almost the same activity as 13-HPODE substrate (Figure S16, Supporting Information). The results indicate that at least two conjugated bonds in the peroxide are required for activity, and the position of the OOH group in the hydrocarbon chain is important. In summary, because bioluminescent activity is strongly influenced by changes in the methyl end (ω-3 vs ω-6 acids and the relative position of the peroxide), we suppose that the substrates interact with the luciferase with this part of the molecule.

### 2.5. Mechanism of Chaetopterus Bioluminescence and Bioimaging of Ferroptosis

Usually, the luciferin molecule contains a system of conjugated bonds representing a chromophore, which participates in the process of light emission. In that case, the fluorescence spectrum of the luciferin oxidation product, namely oxyluciferin, coincides with the bioluminescence spectrum [1]. However, for some glowing organisms, such as bacteria, the mollusc *Latia neritoides* and the worm *Diplocardia longa*, luciferins and oxyluciferins are small organic molecules that do not contain a system of conjugated bonds. In bacteria, the cofactor, a hydroxylated derivative of flavin mononucleotide, acts as a light emitter in the bioluminescence reaction [24]. For *Latia* and *Diplocardia* the light emitters are still unknown. In Siberian earthworms *Henlea* sp. with the luciferin of yet unreported structure, the proposed light emitter (deazaflavin cofactor) is the molecule that is different from it [25]. Neither partially purified *Chaetopterus* luciferase nor the studied substrates are fluorescent in the spectral region near *Chaetopterus* bioluminescence. Thus, the mechanism of light emission in the *Chaetopterus* bioluminescence reaction remains intriguing and is of certain fundamental and practical interest. The studies of the mechanism should include the isolation of the possible oxidation product, the oxyluciferin, which has not yet been performed.

Ferroptosis, a recently discovered type of programmed cell death, is accompanied by uncontrolled iron-mediated lipid peroxidation [26,27]. Accumulation of Fe^2+^ ions and formation of lipid peroxides leading to cell damage is also observed in a number of neurodegenerative diseases, such as Alzheimer’s disease, Parkinson’s disease, Huntington’s disease, Friedreich’s ataxia, and other pathological processes, including amyotrophic lateral sclerosis, periventricular leukomalacia, ischemic stroke, traumatic brain damage, and acute kidney injury. All these health disorders are presumably associated with the launch of the ferroptosis cascade [28]. In order to investigate the mechanisms of ferroptosis in living cells, certain bioimaging strategies, including fluorescent probes for measurement of Fe^2+^, Fe^3+^ ions, ROS (reactive oxygen species), etc., were developed [29,30]. The main disadvantage of these strategies is that they use indirect approaches and could also indicate other pathological processes, such as lipid peroxidation or oxidative stress, but not exactly ferroptosis. In the meantime, *Chaetopterus* luciferase, which utilizes both PUFA or phospholipid hydroperoxides and ferrous ions, might overcome these limitations and become a useful tool for existing bioimaging methods, when the mechanism of luminescence is thoroughly studied and the active recombinant luciferase is available.

In conclusion, we isolated and identified the compounds that demonstrate bioluminescence activity with *Chaetopterus* luciferase. The structures of dienoic acids peroxides and their galactose conjugates were confirmed by using NMR and total synthesis. We also synthesized a number of their structural analogues and showed that *Chaetopterus* luciferase has broad substrate specificity and could catalyze the bioluminescence reaction for peroxides of certain polyunsaturated fatty acids and their derivatives, as well as phospholipid peroxides, in the presence of ferrous ions. However, the mechanism of light emission in *Chaetopterus* bioluminescence reaction is still unknown. Further studies of *Chaetopterus* luciferase are of great interest as they may allow the development of new tools for the bioimaging of ferroptosis in living cells.

## 3. Materials and Methods

### 3.1. Chaetopterus Variopedatus Biomass and Luciferase

*Chaetopterus variopedatus* was collected at two locations: São Sebastião Strait of the coast of Brazil and Trinity bay in the Possiet Gulf of Japan Sea. The worms were pulled out of their tubes and immediately frozen in liquid nitrogen. The frozen polychaetes were shipped on dry ice and stored at –70 °C.

### 3.2. Hitik1, Hitik2, Hitik3 Purification Procedure

Algae of the species *Chaetomorpha linum* from a private marine aquarium were used. An amount of 100 g (wet biomass) of algae *C. linum* were removed from the aquarium and rinsed with distilled water. All further procedures were carried out at 4 °C. The cell fluid from the algae biomass was squeezed out using a manual juicer in order to avoid heating of the resulting extract. Ethyl alcohol, cooled to −20 °C, was added to the obtained extract to a final concentration of 60%. Then, the extract was centrifuged at 25,000× *g* 20 min in an Avanti^®^ J-E centrifuge (Beckman Coulter, Brea, CA, USA). The precipitate was discarded; supernatant was diluted twofold with 10 mM phosphate buffer, pH 9.0. The resulting extract was passed through a 30 × 100 mm Cellulose DEAE 32 column with BioLogic Duo-Flow chromatography system (Bio-Rad Laboratories, Hercules, CA, USA). The filtrate was applied to a 20 × 100 mm column containing the Diasorb-60-C16T sorbent (Biohimmak, Moscow, Russia). The column was equilibrated and washed with a solution of 50% aqueous ethanol. The substance was eluted from the sorbent with 96% ethanol isocratically. Luminescent active fractions were collected, dried on a rotary evaporator, diluted in 400 μL of 96% ethanol, and purified using HPLC. A semi-preparative column (9.4 × 250 mm), ZORBAX Eclipse XDB-C18 (Agilent Technologies, Santa Clara, CA, USA), connected to an Agilent 1260 Infinity LC chromatography system (Agilent Technologies, Santa Clara, CA, USA) was used. Elution was performed using a gradient elution program: solvent A—0.1% formic acid and solvent B—acetonitrile. The standard gradient program was 5–40% B over 25 min, with a flow rate of 3 mL/min. Absorption was monitored at 210, 230, 250, 270, 290, 310, 330, and 360 nm. Fractions, which demonstrated bioluminescence activity and corresponded to peaks 1–3, were collected separately (Figure 1). As a result, three active samples were obtained. The samples were rechromatographed separately on the same column. The resulting active fractions of the peaks were dried on an Eppendorf concentrator 5301rotary evaporator (Eppendorf, Hamburg, Germany) at r. t. and diluted in 100 µL of 96% ethanol, then chromatographed on gel filtration chromatography using Superdex Peptide 10/300 GL column (Cytiva, Marlborough, MA, USA) in a mixture of 0.1% formic acid and 50% acetonitrile. Fractions, corresponding to peaks 1, 2, and 3, named **Hitik1** (2.6 absorbance units), **Hitik2** (2.9 absorbance units), and **Hitik3** (0.15 absorbance units), respectively, which exhibited bioluminescence activity when mixed with partially purified luciferase *C. variopedatus* [9] and ferrous ions Fe^2+^, were collected and dried on a rotary evaporator.

### 3.3. LOX-Catalyzed Peroxidation of PUFA

A solution of linoleic acid (2.8 g/10 mL of ethanol) was mixed with 33 mm phosphate buffer (pH = 6) containing soybean lipoxygenase-1 type-IB, ≥50,000 units/mg (Sigma Aldrich, St. Louis, MO, USA). Oxygen was bubbled through the resulting mixture for 22 h. The products were then extracted by adding of 1 M HCl to pH = 4 and diethyl ether. After shaking the solution, the diethyl ether layer was collected and dried. Products are further purified using column chromatography in the CHCl_3_:Isopropanol 50:1 system. Fractions, containing couples of peroxides 9-(*Z*,*E*)-HPODE and 9-(*E*,*E*)-HPODE, 13-(*Z*,*E*)-HPODE and 13-(*E*,*E*)-HPODE, were further purified using RP-HPLC on PuriFlash 5.250 system with UV detector (Interchim, Montluçon, France) with 100 × 21.2 mm Gemini 10 um C18 column (Phenomenex, Torrance, CA, USA) eluted with an acetonitrile/water linear gradient—50–100% acetonitrile, flow rate = 10 mL/min, λ = 235 nm.

### 3.4. Methylene Blue Catalyzed Photo-Oxidation of PUFA or Their Derivatives

PUFA and their derivatives (0.1 mmol) were dissolved in 1 mL of methanol and the resulting solution was placed in an ampoule. A solution of methylene blue in methanol was added at a concentration of 0.1 mg/mL (0.13 mmol). A 150 W incandescent lamp (Nova II) was placed at a distance of 15–20 cm in front of the ampoule, and oxygen was bubbled through the reaction mixture for 4–6 h until TLC (thin layer chromatography) (CH_2_Cl_2_: Isopropanol, 95:5) showed complete conversion of the starting PUFA and their derivatives into more polar products. Products were further purified using RP-HPLC on the PuriFlash 5.250 system with UV-detector (Interchim, Montluçon, France) with 150 × 10 mm Luna 10 um C18(2) column (Phenomenex, Torrance, CA, USA) eluted with acetonitrile/water linear gradient—50–100% acetonitrile, flow rate = 5 mL/min, λ = 235 nm.

### 3.5. Bioluminescence Spectrum of 13-HPODE

A 50 μL aliquot of the partially purified *Chaetopterus* enzyme [9] was incubated for 10 min in the presence of 2 mM 13-HPODE in 200 μL of 50 mM phosphate buffer (pH 8.0). Then, the sample was placed in a cuvette within a Shimadzu-UV-Mini-1240-UV-VIS Spectrophotometer (Shimadzu, Kyoto, Japan). A solution of FeSO_4_ was continuously pumped into the cuvette at a rate of 0.25 mM / min, and the chemiluminescent visible light spectrum was measured from the moment pumping began for up to 5 min or until the luminescent reaction was below the level of detection for the spectrophotometer.

### 3.6. Bioluminescence Activity Assay

Reactions were monitored with a custom made luminometer BLM (Oberon-K, Krasnoyarsk, Russia). For each measurement 100 μL of reaction mixture (phosphate-buffered saline (pH 7.4)), 1 μL of luciferase fraction, 1 μL of 3 mM peroxide methanolic solution, and 1 μL 10 mM FeSO_4_ solution) were used. Measurements were corrected for background luminescence of PUFA peroxides in the presence of Fe^2+^ ions. All experiments were performed in triplicate. The data are expressed as mean ± SD (n = 3) and analyzed using Student’s t-test. Differences were considered significant at *p* < 0.05.

### 3.7. Other Methods, Synthetic Procedures, and NMR Spectra

Details of the other materials and methods used in this study, including procedures to obtain synthetic compounds and their characterization using MS and NMR (Figures S3–S12) are provided in Supporting Information.

## Data Availability

The data presented in this study are available in the Supporting Information file. Additional data are available from the corresponding author on reasonable request.

## Supplementary Materials

The supporting information can be downloaded at https://www.mdpi.com/article/10.3390/ijms24119466/s1.
