# Supplementary material for: Conjugated Dienoic Acid Peroxides as Substrates in Chaetopterus Bioluminescence System
Source: Int J Mol Sci. 2023 May 30;24(11):9466. doi: 10.3390/ijms24119466 (PMC10253912; doi:10.3390/ijms24119466)
Supplement: Supplementary file 1 [file ijms-24-09466-s001.zip › ijms-2377845-supplementary.pdf]

Supporting information for

Conjugated dienoic acid peroxides as substrates in *Chaetopterus* bioluminescence system

Renata I. Zagitova, Konstantin V. Purtov, Aleksandr S. Shcheglov, Konstantin S. Mineev, Maxim A. Dubinnyi, Ivan N. Myasnyanko, Olga A. Belozerova, Vera G. Pakhomova, Valentin N. Petushkov, Natalia S. Rodionova, Vladislav A. Lushpa, Elena B. Guglya, Sergey Kovalchuk, Valeri B. Kozhemyako, Jeremy D. Mirza, Anderson G. Oliveira, Ilia V. Yampolsky, Zinaida M. Kaskova, Aleksandra S. Tsarkova

Contents

Materials and methods **2**
NMR data of luminescent compounds from *Chaetomorpha linum* **3**
Synthesis and purification of synthetic compounds **7**
NMR data of synthetic compounds **19**
Bioluminescence activity assay **31**

Substance numbering in Supporting information is independent from the main text.

Materials and methods

Chemicals and synthesis. 1-palmitoyl-2-linoleoyl-sn-glycero-3-phosphocholine (PLPC) was obtained from Avanti Polar Lipids (Alabaster, Alabama, USA). Linoleic acid (for synthesis) was obtained from Merck KGaA (Darmstadt, Germany). Other chemicals, unless otherwise stated, were from Sigma-Aldrich (St. Louis, MO, USA) and the purest grade available. Merck Kieselgel 60 was used for column chromatography. Thin layer chromatography was performed on silica gel 60 F254 aluminium plates (Merck). Visualization was effected by UV light (254 nm) and staining with $KMnO_4$ or ceric ammonium molybdate solutions.

NMR analysis. All NMR spectra of natural compounds were recorded on the Avance III Bruker 800 spectrometer (Bruker Biospin, Germany), with the magnetic field strength for protons equal to 800 MHz. The spectrometer was equipped with the triple resonance cryogenic TCI probe with cold ^{13}C preamplifier. All spectra were recorded at 30ºC in DMSO-d_6 (Cambridge Isotope Laboratories, USA). To measure the *J*-couplings across the double bonds, we implemented, if necessary, the double resonance experiments were employed.

NMR spectra of synthetic compounds were acquired in DMSO-d_6, $CDCl_3$ (Eurisotop, France) at 30°C with Me_4Si as an internal standard on the Bruker Fourier 300 MHz and Avance III Bruker 600 MHz, 700 MHz and 800 MHz spectrometers.

HRMS analysis. HRMS spectra of natural compounds isolated from *Chaetomorpha linum* were obtained on an Agilent 6224 TOF LC/MS System (Agilent Technologies, Santa Clara, CA, USA) equipped with a YMC-Triart C18 column (3 μm, 12 nm, 75 × 4.6 mm) and dual-nebulizer ESI source. Samples were eluted with a H_2O-MeCN gradient (from 5 to 50% of MeCN) with 0.1% trifluoroacetic acid as an eluent additive. Data acquisition and analysis was performed by the MassHunter Workstation software (Agilent Technologies, Santa Clara, CA, USA).

HRMS spectra of synthetic compounds were obtained on an Ultimate 3000 RSLCnano HPLC system (Thermo Scientific™) equipped with a Luna Omega C18 column (2.5 μm, 100 Å, 100 x 2.1 mm) and Orbitrap Fusion Lumos mass spectrometer (Thermo Scientific™). Samples were eluted with a H_2O-MeCN linear gradient (from 30 to 95% of MeCN, 150 ul/min) with 10 mM ammonium formate and 0.1% formic acid as an eluent additive. UV data was collected at 235 nm. MS1 and MS2 spectra were recorded at 30K and 15K resolution respectively with HCD fragmentation. Data acquisition and analysis was performed by the Xcalibur™ Software (Thermo Scientific™).

LC-MS analysis of synthetic compounds was carried out on ACQUITY UPLC H-Class System (Waters Corporation, USA) equipped with a ACQUITY UPLC BEH C18 Column (1.7 μm, 130Å, 50 mm X 2.1 mm) and ESI source. Samples were eluted with a H_2O-MeCN linear gradient (from 5 to 95% of MeCN, 150 ul/min) with 0.1% formic acid as an eluent additive. UV data was collected at 220 nm.

NMR data of luminescent compounds from *Chaetomorpha linum*

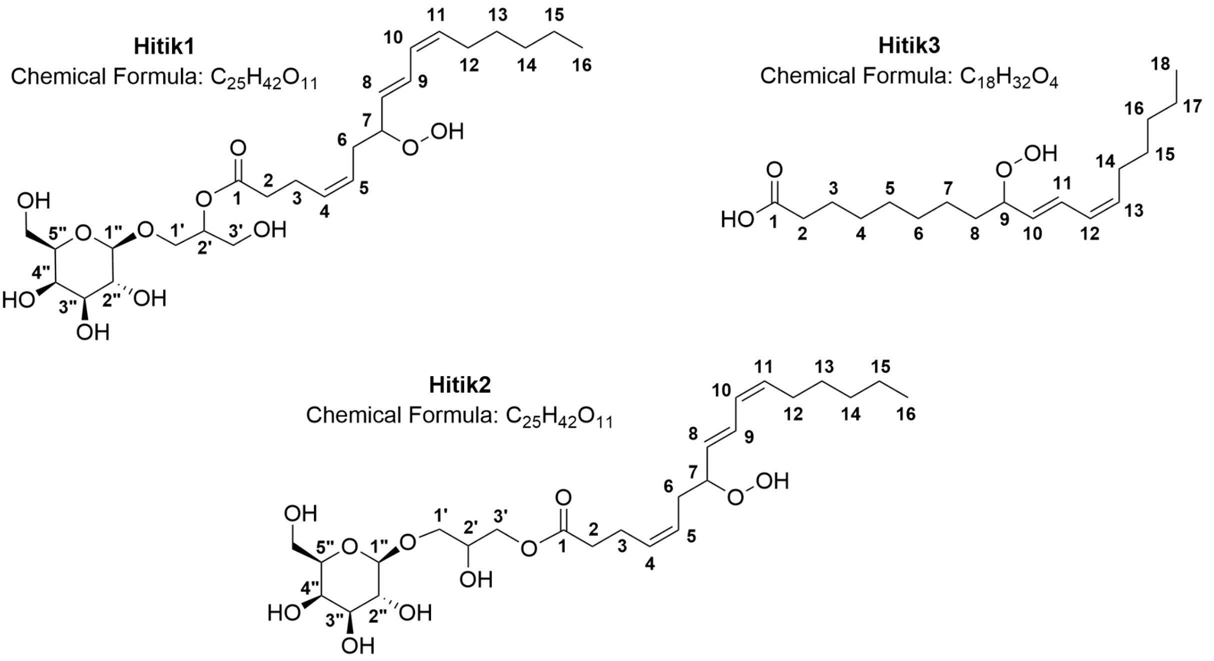

Figure S1. Structures of *Chaetopterus* luciferase substrates isolated from algae *Chaetomorpha linum* according to NMR and HRMS data.

Table S1. Chemical shifts and relevant NMR data for the compound **Hitik1**.

Position	^{13}C	^{1}H	multiplicity	HMBC/TOCSY
**1**	172.56	-		
**2**	33.96	2.36	dt(3,7)[a]	1,3,4/3[b]
**3**	22.94	2.26	ov.[c]	1,2,4,5/2,4
**4**	130.26	5.44	ov.	3,6/3,5
**5**	126.19	5.37	m(11 to 5,DR)[d]	3,6/4,6
**6**	30.93	2.27;2.42	ov./ov.	4,5,7,8/5,7
**7**	84.64	4.29	q(7)	5,6,8,9/6,8
**7-OOH**	-	11.40	s	-
**8**	132.73	5.61	dd(15,7)	6,7,10/7,9
**9**	128.37	6.49	dd(15,11)	7,10,11/8,10
**10**	128.49	5.98	dd(11,11)	8,9,12/9,11
**11**	133.03	5.46	ov.	9,12/10,12
**12**	27.55	2.17	q(7)	10,11,13,14/11,13
**13**	29.17	1.37	p(7)	11,12,14,15/12,14

**14**	31.27	1.28	ov.	
**15**	22.40	1.30	ov.	
**16**	14.39	0.87	t(7)	14,15/15
**1'**	67.57	3.61;3.79	dd(6,11)/dd(6,11)	**1"**,2',3'/2'
**2'**	74.01	4.89	p(7)	1',3'/1',3'
**3'**	60.30	3.57;3.63	ov.	**1**,1',2'/2'
**3'-OH**		4.78	br.	-/3'
**1"**	104.25	4.09	d(7)	1',2",3"/2"
**2"**	70.93	3.29	ov.	-/1",3"
**2"-OH**		4.79	br.	-/2"
**3"**	73.88	3.28	ov.	-/2",4"
**3"-OH**		4.70	br.	-/3"
**4"**	68.60	3.64	m.	3"/3"
**4"-OH**		4.34	d(4)	3",4",5"/4"
**5"**	75.81	3.34	ov.	3",4",6",**1"**/6"
**6"**	60.85	3.49;3.51	ov.	-/5"
**6"-OH**		4.53	br.	-/6"

[a]multiplicity of the signal (s - singlet, d - doublet, t - triplet, q - quadruplet, p - quintet, m - complex multiplet)
[b]correlations between the current proton and carbons in HMBC/HSQC-TOCSY (mixing time=10 ms) spectra
[c]ov. - overlapped signals, br. - broad signal

Table S2. Chemical shifts, and relevant NMR data for the compound **Hitik2**.

Position	^{13}C	^{1}H	multiplicity	HMBC/TOCSY
**1**	172.84	-		
**2**	33.79	2.36	dt(3,7)[a]	1,3,4/3[b]
**3**	22.94	2.26	ov.[c]	1,2,4,5/2,4
**4**	130.26	5.42	ov.	3,6/3,5
**5**	126.19	5.37	m(11 to 5,DR)[d]	3,6/4,6
**6**	30.93	2.27;2.42	ov./ov.	4,5,7,8/5,7
**7**	84.64	4.30	q(7)	5,6,8,9/6,8
**7-OOH**	-	11.40	s	-
**8**	132.73	5.61	dd(15,7)	6,7,10/7,9

**9**	128.37	6.49	dd(15,11)	7,10,11/8,10
**10**	128.49	5.98	dd(11,11)	8,9,12/9,11
**11**	133.03	5.46	ov.	9,12/10,12
**12**	27.55	2.17	q(7)	10,11,13,14/11,13
**13**	29.17	1.37	p(7)	11,12,14,15/12,14
**14**	31.27	1.28	ov.	
**15**	22.40	1.30	ov.	
**16**	14.39	0.87	t(7)	14,15/15
**1'**	70.88	3.44;3.70	ov.	**1"**,2',3'/2'
**2'**	67.88	3.85	m	1',3'/1',3'
**2'-OH**		4.97	d(5.5)	1',2',3'/2'
**3'**	66.09	3.99;4.07	dd(7,11)/ov.	1,1',2'/2'
**1"**	104.25	4.09	d(7)	**1'**,2",3"/2"
**2"**	71.01	3.29	ov.	-/1",3"
**2"-OH**		4.85	d(4)	1",2",3"/2"
**3"**	73.75	3.28	ov.	-/2",4"
**3"-OH**		4.69	d(4.5)	2",3",4"/3"
**4"**	68.60	3.64	m.	3"/3"
**4"-OH**		4.36	d(4)	3",4",5"/4"
**5"**	75.81	3.34	ov.	3",4",6",1"/6"
**6"**	60.88	3.48;3.53	ov.	-/5"
**6"-OH**		4.56	d(4.5)	5",6"/6"

[a]multiplicity of the signal (s - singlet, d - doublet, t - triplet, q - quadruplet, p - quintet, m - complex multiplet)
[b]correlations between the current proton and carbons in HMBC/HSQC-TOCSY (mixing time=10 ms) spectra
[c]ov. - overlapped signals, br. - broad signal
[d]DR - J-coupling measured in double resonance experiment

Table S3. Chemical shifts and relevant NMR data for the compound **Hitik3**.

Position	^{13}C	^{1}H	multiplicity	HMBC/TOCSY
**1**	174.89	-		
**1-OH**	-	11.92	br.[a]	-
**2**	34.16	2.18	t(7)[c]	1,3,4/3[b]

**3**	24.95	1.49	p(7)	1,2,4,5/2,4
**4**	28.97	1.26	ov.	-
**5**	29.13	1.26	ov.	-
**6**	29.32	1.26	ov.	-
**7**	25.20	1.26	ov.	-
**8**	32.78	1.39;1.59	m/m	6,7,9,10/7,9
**9**	85.08	4.24	q(7)	7,8,10,11/8,10
**9-OOH**	-	11.28	s	-
**10**	133.71	5.60	dd(15,7)	8,12/9,11
**11**	127.97	6.47	dd(15,11)	9,12,13/10,12
**12**	128.61	5.99	dd(11,11)	10,11,14/11,13
**13**	132.66	5.44	dt(11,7)	11/12,14
**14**	27.51	2.15	q(7)	12,13,15,16/13,15
**15**	29.17	1.35	p(7)	13,14,16,17/14,16
**16**	31.24	1.26	ov.	-
**17**	22.43	1.28	ov.	-
**18**	14.39	0.87	t(7)	16,17/17

[a]ov. - overlapped signals, br. - broad signal

[b]correlations between the current proton and carbons in HMBC/HSQC-TOCSY (mixing time=10 ms) spectra

[c]multiplicity of the signal (s - singlet, d - doublet, t - triplet, q - quadruplet, p - quintet, m - complex multiplet)

Synthesis and purification of synthetic compounds

(10*E*,12*Z*)-9-hydroperoxyoctadecaenoic acid (1) and (9*Z*,11*E*)-13-hydroperoxy-octadecaenoic acid (2).

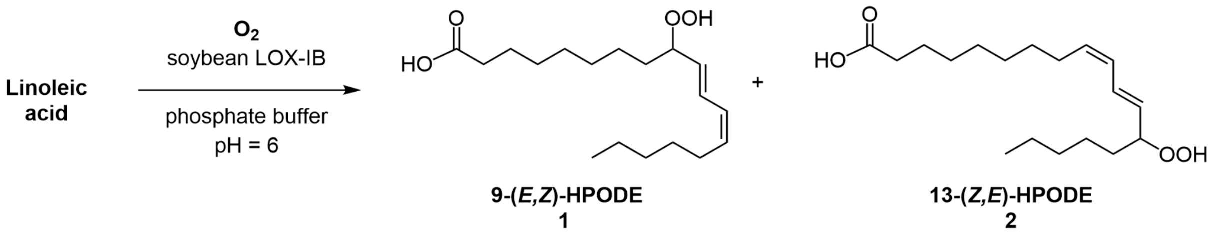

Solution of 2.78 g of linoleic acid (10 mmol) in 10 ml of methanol was mixed with 1 l of 33 mm phosphate buffer (pH=6) and vigorously stirred to obtain a homogeneous emulsion. Then 30 mg soybean lipoxygenase LOX-IB (Sigma Aldrich, type-IB, ≥ 50,000 units/mg) was added. Oxygen was bubbled through the resulting mixture. The progress of the reaction was monitored by TLC ($CHCl_3$:IPA 20:1). After 22 hours the conversion stopped changing, 1M HCl was added to the reaction mixture to pH=4 and products were extracted with diethyl ether (3 x 600 ml). The combined organic phase was dried over anhydrous Na_2SO_4, the solvent was evaporated. Crude mixture consisted of unreacted linoleic acid (38%), 9-(*E,Z*)-HPODE (**1**), 13-(*Z,E*)-HPODE (**2**), and other impurities formed by unselective oxidation. The products are further purified using column chromatography in the $CHCl_3$:IPA 50:1 system. Fractions, containing **1** and **2**, were further purified using RP-HPLC on PuriFlash 5.250 system with UV-detector (Interchim, France) with 100 x 21.2 mm Gemini 10 um C18 column (Phenomenex, Torrance, CA) eluted with acetonitrile/water linear gradient – 50-100% acetonitrile, flow rate = 10 mL/min, λ = 235 nm. Products **1** and **2** were obtained as colourless oils - 98 mg (10%) and 137 (14%) mg respectively.

(10E,12Z)-9-hydroperoxyoctadecaenoic acid:

^{1}H NMR (700 MHz, DMSO-d_6): δ 11.91 (s, 1H), 11.26 (s, 1H), 6.46 (dd, *J* = 15.3, 11.1 Hz, 1H), 5.98 (td, *J* = 11.0, 1.6 Hz, 1H), 5.59 (dd, *J* = 15.3, 7.7 Hz, 1H), 5.43 (dt, *J* = 11.0, 7.7 Hz, 1H), 4.23 (q, *J* = 6.8 Hz, 1H), 2.18 (t, *J* = 7.5 Hz, 2H), 2.15 (q, *J* = 7.5 Hz, 2H), 1.61 – 1.54 (m, 1H), 1.48 (p, *J* = 7.2 Hz, 2H), 1.42 – 1.37 (m, 1H), 1.34 (p, *J* = 7.3 Hz, 2H), 1.30 – 1.22 (m, 12H), 0.86 (t, *J* = 7.0 Hz, 3H)

^{13}C NMR (176 MHz, DMSO-d_6): δ 174.91, 133.73, 132.65, 128.59, 127.96, 85.10, 34.13, 32.80, 31.25, 29.33, 29.19, 29.14, 28.96, 27.52, 25.20, 24.95, 22.39, 14.36.

HRESIMS m/z calcd for $C_{18}H_{31}O_3^+$ [M-H_2O+H]$^+$ - 295.2268, found - 295.2268, calcd for $C_{18}H_{32}O_4Na^+$ [M + Na]$^+$ - 335.2193, found - 335.2195.

(9Z,11E)-13-hydroperoxyoctadecaenoic acid

^{1}H NMR (700 MHz, DMSO-d_6): δ 11.92 (s, 1H), 11.26 (s, 1H), 6.46 (dd, *J* = 15.3, 11.1 Hz, 1H), 5.98 (td, *J* = 11.0, 1.6 Hz, 1H), 5.59 (dd, *J* = 15.3, 7.7 Hz, 1H), 5.42 (dt, *J* = 10.8, 7.6 Hz, 1H), 4.23 (q, *J* = 6.8 Hz, 1H), 2.18 (t, *J* = 7.4 Hz, 2H), 2.15 (q, *J* = 7.5 Hz, 2H), 1.59 – 1.55 (m, 1H), 1.48 (p, *J* = 7.2 Hz, 3H), 1.42 – 1.36 (m, 0H), 1.33 (p, *J* = 6.8 Hz, 2H), 1.29 – 1.22 (m, 12H), 0.85 (t, *J* = 7.0 Hz, 3H).

^{13}**C NMR** (176 MHz, DMSO-d_6): δ 174.94, 133.76, 132.64, 128.61, 128.00, 85.13, 34.15, 32.78, 31.69, 29.48, 29.04, 29.01, 28.90, 27.55, 24.97, 24.91, 22.49, 14.34.

HRESIMS m/z calcd for $C_{18}H_{31}O_3^+$ [M-H_2O+H]$^+$ - 295.2268, found - 295.2268, calcd for $C_{18}H_{32}O_4Na^+$ [M + Na]$^+$ - 335.2193, found - 335.2195.

9,10,12- and 13-hydroperoxides of octadecadienoic acid (1-4).

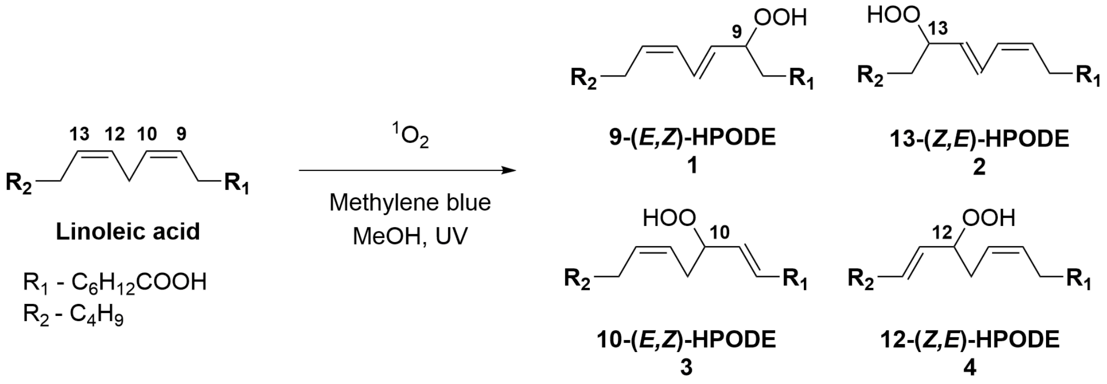

The preparation of peroxides from 30 mg (0.1 mmol) of linoleic acid was carried out according to the methylene blue catalyzed photo-oxidation procedure described above; 6 mg (0.02 mmol) of a mixture of products **1-4** (0.05 mmol, 20% yield) was obtained.

^{1}**H NMR** (800 MHz, $CDCl_3$): δ 6.57 (ddd, J = 15.1, 10.9, 4.0 Hz, 1H), 6.01 (t, J = 11.3 Hz, 1H), 5.77 (tq, J = 14.0, 6.9 Hz, 1H), 5.57 (ddd, J = 14.0, 8.1, 4.6 Hz, 1H), 5.54 – 5.45 (m, 2 x 1H), 5.43 – 5.37 (m, 1H), 5.34 (q, J = 9.2, 8.7 Hz, 1H), 4.38 (p, J = 7.0 Hz, 1H), 4.31 (q, J = 7.2 Hz, 1H), 2.34 (q, J = 6.2, 5.0 Hz, 2 x 2H), 2.18 (dd, J = 13.8, 6.6 Hz, 2H), 2.08 (p, J = 6.6, 5.7 Hz, 1H), 2.03 (dd, J = 14.2, 7.1 Hz, 1H), 1.68 – 1.59 (m, J = 7.8, 7.4 Hz, 2 x 3H), 1.36 – 1.27 (m, 2 x 15H), 0.89 (t, J = 6.2 Hz, 2 x 3H).

9- and 10-octadecaenoic acid hydroperoxides (5-6).

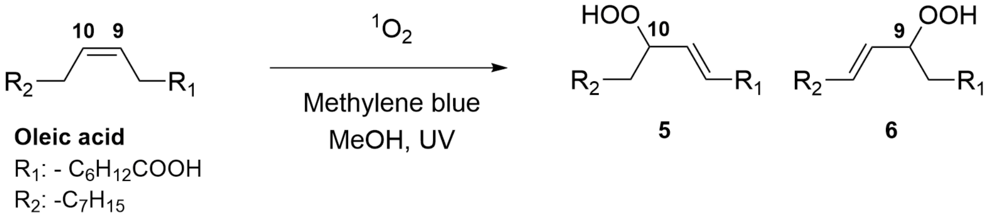

Obtaining peroxides **5-6** from 30 mg (0.1 mmol) of oleic acid was carried out according to the methylene blue catalyzed photo-oxidation procedure described above, 3 mg (0.01 mmol, 10%) of a mixture of 2 isomeric products was obtained.

^{1}**H NMR** (700 MHz, $CDCl_3$): δ 6.89 (ddt, J = 16.0, 9.0, 6.9 Hz, 1H), 5.83 (dq, J = 14.5, 7.2, 6.8 Hz, 1H), 5.48 – 5.40 (m, 2 x 1H), 4.34 (q, J = 7.2 Hz, 2 x 1H), 2.59 (t, J = 7.5 Hz, 2 x 2H), 2.38 (q, J = 7.5 Hz, 2 x 2H), 2.16 (q, J = 7.4 Hz, 2H), 2.09 (q, J = 6.8 Hz, 2H), 1.73 – 1.66 (m, 2 x 2H), 1.66 – 1.61 (m, 2 x 2H), 1.54 (q, J = 7.1 Hz, 2 x 2H), 1.48 (p, J = 7.3 Hz, 2 x 2H), 1.44 – 1.30 (m, 2 x 12H), 0.98 – 0.94 (m, 2 x 3H).

9,10,12,13,15,16-hydroperoxides of octadecatrienoic acid (7-12).

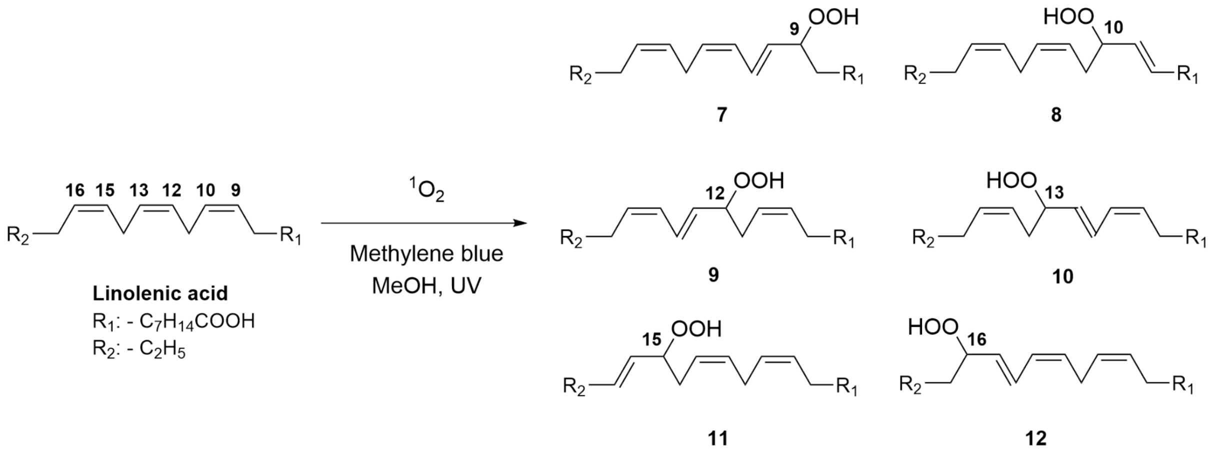

The preparation of peroxides **7-12** from 30 mg (0.1 mmol) of linolenic acid was carried out according to the methylene blue catalyzed photo-oxidation procedure described above; 4 mg (0.01 mmol, 12% yield) of a mixture of products was obtained.

^{1}H NMR (800 MHz, $CDCl_3$): δ 6.59 (dtt, J = 15.3, 10.8, 5.3 Hz, 2 x 1H), 6.00 (dq, J = 26.4, 14.5, 12.8 Hz, 3 x 1H), 5.84 – 5.73 (m, 3 x 1H), 5.59 (dt, J = 14.5, 7.1 Hz, 2 x 1H), 5.53 – 5.46 (m, 3 x 1H), 5.44 – 5.32 (m, 5H), 4.43 (q, J = 7.0 Hz, 1H), 4.39 (q, J = 7.1 Hz, 1H), 4.34 (q, J = 7.2 Hz, 1H), 2.95 (t, J = 7.2 Hz, 1H), 2.81 – 2.77 (m, 1H), 2.35 (q, J = 6.5, 6.1 Hz, 3 x 2H), 2.30 (p, J = 7.0, 6.5 Hz, 2H), 2.23 – 2.15 (m, 3H), 2.11 – 2.01 (m, 3 x 2H), 1.64 (t, J = 7.4 Hz, 8H), 1.37 – 1.28 (m, 25H), 1.01 (t, J = 7.7 Hz, 3H), 0.97 (t, J = 6.5 Hz, 3H), 0.94 (t, J = 7.2 Hz, 3H), 0.91 – 0.87 (m, 3H).

1,2,3,4,6-penta-O-acetyl-D-galactopyranose (13).

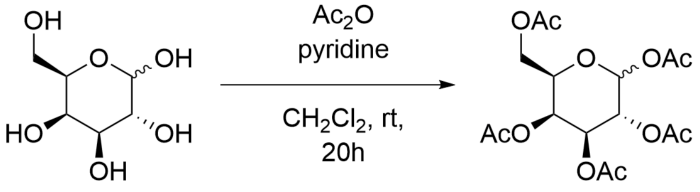

13

15 mL of acetic anhydride was added to a flask and was carefully mixed with 2.5 g of D-galactose. While stirring with a magnetic stirrer, anhydrous pyridine (19 mL) was added. This mixture was kept at room temperature under stirring for 20 h. When the reaction was completed, the solution was mixed with ice-cold water and washed with EtOAc (3 x 100 ml). The organic phase was washed successively with saturated $NaHCO_3$ solution and water, dried over anhydrous Na_2SO_4. The solvent was evaporated, the substance was isolated by column chromatography (Hex:EtOAc 1:1, Rf = 0.43): colorless, sticky solid (4.6 g, 85%).

^{1}H NMR (300 MHz, $CDCl_3$): δ 6.33 (d, 1Hα, *J* = 1.5 Hz, H-1α), 5.68 (d, 1Hβ, *J* = 8.3 Hz, H-1β), 5.40 (dd, *J* = 3.4, 1.1 Hz, 1H), 5.31 (t, *J* = 1.5 Hz, 2H), 5.06 (dd, *J* = 10.4, 3.4 Hz, 1H), 4.32 (td, *J* = 6.7, 1.4 Hz, 1H), 4.16-4.01 (m, 2H,), 2.14, 2.10, 2.02, 2.00, 1.98, 1.97 (all s, 15H, 5×C(O)CH_3).

2,3,4,6-tetra-O-acetyl-α-D-galactopyranosyl bromide (14).

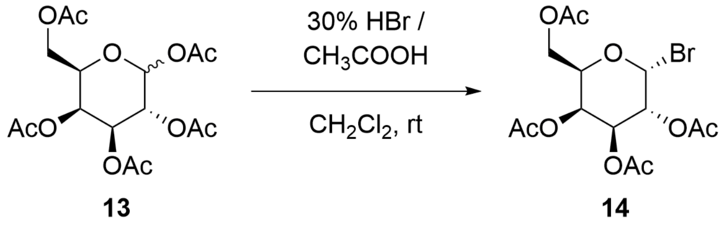

A solution of **13** (1.5 g, 3.85 mmol) in a mixture of DCM (5 ml) and 30% HBr/AcOH (10 ml) was stirred at room temperature until TLC (Hex:EtOAc 2:1) showed complete conversion to a 2 faster moving products (Rf = 0.32, α-anomer and Rf = 0.42, β-anomer). The solution was diluted with 20 ml of DCM and washed successively with ice-cold water and saturated $NaHCO_3$ solution, dried over anhydrous Na_2SO_4. The solvent was evaporated, the target product **14** was isolated by column chromatography (Hex:EtOAc 2:1): yellow, sticky solid (1.06 g, 67%).

^{1}H NMR (300 MHz, $CDCl_3$): δ 6.69 (d, *J* = 3.9 Hz, 1H), 5.52 (dd, *J* = 3.3, 1.3 Hz, 1H), 5.40 (dd, *J* = 10.6, 3.3 Hz, 1H), 5.05 (dd, *J* = 10.6, 4.0 Hz, 1H), 4.51 – 4.45 (m, 1H), 4.21 – 4.07 (m, 2H), 2.15, 2.11, 2.06, 2.01 (all s, 12H, 4×C(O)CH_3).

1,2-O-isopropylidene-rac-glycerol (15).

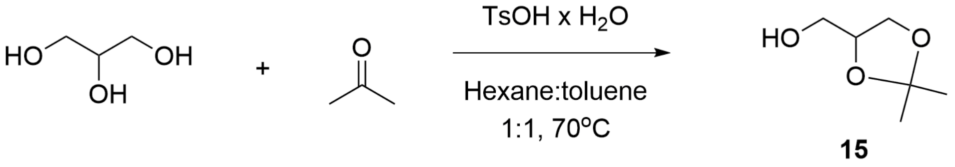

To a 250 ml three-necked round bottom flask equipped with a condenser and a Dean-Stark apparatus was added glycerol (17.0 g, 185 mmol), acetone (50 ml), hexane (150 ml), toluene (150 ml) and PTSA x 2H2O (0.512 g , 2.69 mmol, 0.015 eq.). The mixture was stirred for 7 hours at 70°C, cooled to room temperature, the solvent was evaporated. The crude product **15** was flash chromatographed with silica gel and Et_2O as eluent. After evaporation of the solvent, the product **15** was obtained as colorless liquid (17.5 g, 73%), which was used in the next step without further purification.

^{1}H NMR (700 MHz, $CDCl_3$): δ 4.22 (tdd, J = 6.5, 5.2, 3.8 Hz, 1H), 4.02 (dd, J = 8.2, 6.5 Hz, 1H), 3.77 (dd, J = 8.2, 6.5 Hz, 1H), 3.70 (dd, J = 11.7, 3.8 Hz, 1H), 3.58 (dd, J = 11.7, 5.3 Hz, 1H), 2.16 (br s, 1H), 1.42 (s, 3H), 1.36 (s, 3H).

^{13}C NMR (176 MHz, $CDCl_3$): δ 109.45, 76.22, 65.77, 63.06, 26.73, 25.30.

1-O-(2', 3', 4', 6'-Tetra-O-acetyl-β-D-galactopyranosyl)-rac-glycerol (16).

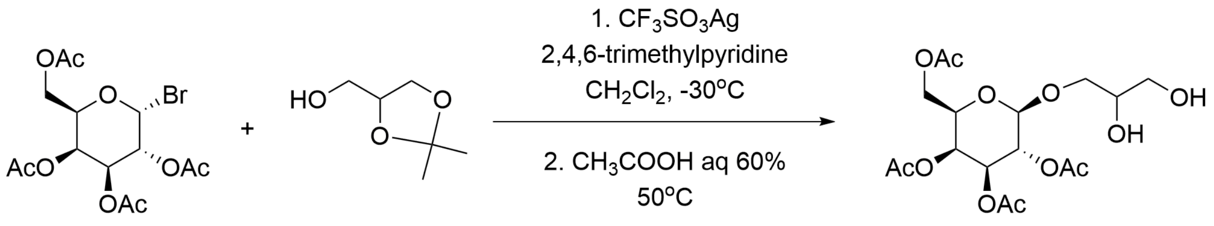

14 **15** **16**

A solution of **14** (1.36 mg, 3 mmol) and 2,4,6-trimethylpyridine (360 mg, 3 mmol) in dichloromethane (5 ml) was added at - 30°C to a suspension of silver trifluoromethanesulfonate (1.09 g, 4.2 mmol) and **15** (0.65 g, 4.9 mmol) in dichloromethane (15 ml), and the mixture was stirred until TLC (Hex:EtOAc 2:1, Rf = 0.26) showed complete conversion into two slower moving products (1 h). The mixture was warmed to room temperature, neutralized by addition of a few drops of 2,4,6-trimethylpyridine and washed with aqueous sodium thiosulfate. The organic phase was evaporated, the resulting mixture was dissolved in 60% AcOH (50 ml) and stirred for 3 hours at 70°C. The reaction mixture was co-evaporated with toluene 3 times for complete removal of acetic acid and water, the product **16** was isolated using column chromatography (Tol:acetone 4:1, Rf=0.3). After evaporation of the solvent, the product **16** was obtained as yellow sticky solid (700 mg, 38%).

^{1}H NMR (700 MHz, $CDCl_3$): δ 5.45 (dt, J = 3.1, 1.4 Hz, 1H), 5.25 (ddd, J = 10.3, 8.0, 2.2 Hz, 1H), 5.09 (dd, J = 10.5, 3.4 Hz, 1H), 4.56 (d, J = 8.0 Hz, 1H), 4.24 – 4.17 (m, 2H), 4.00 (tdd, J = 6.9, 3.4, 1.2 Hz, 1H), 3.93 – 3.89 (m, 1H), 3.87 – 3.80 (m, 1H), 3.79 – 3.71 (m, 2H), 3.71 – 3.64 (m, 1H), 2.22 (s, 3H), 2.13 (s, 3H), 2.12 (s, 3H), 2.04 (s, 3H).

1-glycerol-2', 3', 4', 6'-tetra-O-acetyl-β-D-galactopyranoside-(9*Z*,12*Z*)-octadecadienoate (17).

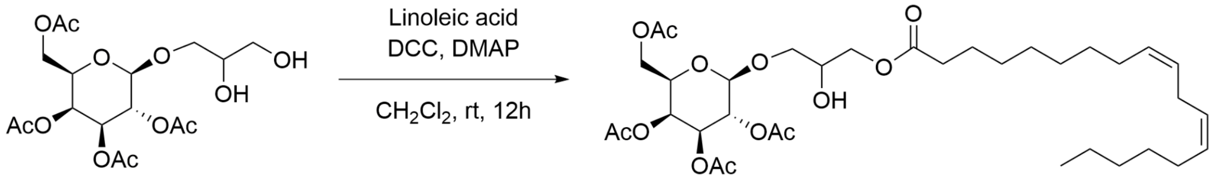

16 **17**

DCC (161 mg, 0.78 mmol) and DMAP (9 mg, 0.07 mmol) were added to a solution of the linoleic acid (200 mg, 0.71 mmol) in 5 ml DCM, and the mixture was stirred for half an hour in an inert atmosphere. Then, a solution of **16** (300 mg, 0,71 mmol) in 2 ml DCM was added dropwise to the mixture and left stirred overnight at room temperature. The precipitate that formed was filtered off, the solvent was evaporated, and the product **17** was isolated using column chromatography Hex:EtOAc 2:1 → Hex:EtOAc 1:1: colorless oily liquid (359 mg, 73%).

^{1}H NMR (700 MHz, $CDCl_3$): δ 5.48 – 5.46 (m, 2 x 1H), 5.45 – 5.38 (m, 2 x 4H), 5.28 (ddd, J = 10.7, 8.0, 2.9 Hz, 2 x 1H), 5.10 (ddd, J = 10.5, 3.4, 1.2 Hz, 2 x 1H), 4.59 (d, J = 8.0 Hz, 1H), 4.58 (d, J = 8.0 Hz, 1H), 4.24 – 4.21 (m, 2 x 1H), 4.21 – 4.19 (m, 2 x 1H), 4.19 – 4.16 (m, 2 x 1H), 4.07 (dq, J = 24.3, 4.8 Hz, 2 x 1H), 4.01 (tdd, J = 6.7, 3.9, 1.2 Hz, 2 x 1H), 3.97 (dd, J = 10.6, 3.6 Hz, 2 x 1H), 3.88 – 3.82 (m, 2 x H), 3.75 (dd, J = 10.6, 6.0 Hz, 2 x 1H), 2.84 (t, J = 7.0 Hz, 2 x 2H), 2.41 (td, J = 7.6, 1.6 Hz, 2 x 2H), 2.24 (s, 2 x 3H), 2.15 (s, 2 x 3H), 2.14-2.10 (m, 2 x 4H), 2.13 (s, 2 x 3H), 2.06 (s, 2 x 3H), 1.69 (dd, J = 15.4, 6.9 Hz, 2 x 2H), 1.43 (q, J = 6.3, 5.6 Hz, 2 x 4H), 1.40 – 1.34 (m, 2 x 11H), 0.96 (t, J = 7.0 Hz, 2 x 3H).

^{13}C NMR (176 MHz, $CDCl_3$): δ 170.42, 130.28, 130.06, 128.14, 127.95, 101.98, 72.17, 71.87, 71.01, 70.81, 68.99, 68.90, 68.83, 67.02, 67.00, 65.10, 61.42, 34.16, 34.00, 31.57, 29.64, 29.38, 29.21, 29.15, 27.25, 27.23, 27.21, 25.68, 24.98, 24.93, 22.61, 20.77, 20.66, 20.58, 14.10.

1-glycerol-β-D-galactopyranoside-(*9Z,12Z*)-octadecadienoate (18).

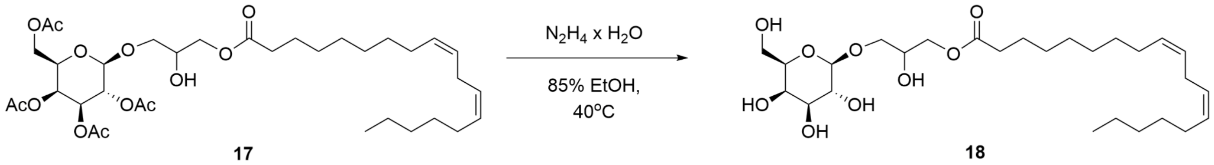

To a solution of **17** (183 mg, 0.3 mmol) in 10 ml of 85% ethanol was added hydrazine monohydrate (128 mg, 2.6 mmol). The resulting mixture was stirred at 40°C until TLC ($CHCl_3$:EtOH, 4:1) showed complete conversion to a more polar product (Rf = 0.22), then the solution was poured into ice water (50 ml) and extracted with chloroform (3 x 20 ml). The combined organic phase was dried over anhydrous Na_2SO_4, the solvent was evaporated, the product **18** was isolated using column chromatography ($CHCl_3$:EtOH 9:1 → $CHCl_3$:EtOH 7:3): colorless oily liquid (55 mg, 35%).

^{1}H NMR (700 MHz, DMSO-d_6): δ 5.39 – 5.28 (m, 4H), 4.94 (dd, J = 10.4, 5.3 Hz, 1H), 4.82 (t, J = 3.8 Hz, 1H), 4.67 (d, J = 5.4 Hz, 1H), 4.54 (td, J = 5.7, 2.6 Hz, 1H), 4.33 (dd, J = 4.6, 2.2 Hz, 1H), 4.14 – 4.03 (m, 2H), 3.97 (ddd, J = 17.8, 11.2, 6.4 Hz, 1H), 3.82 (ddt, J = 9.9, 6.2, 3.6 Hz, 1H), 3.70 (ddd, J = 10.4, 6.7, 5.4 Hz, 1H), 3.63 (t, J = 4.0 Hz, 1H), 3.54 (dt, J = 11.7, 6.1 Hz, 1H), 3.48 (q, J = 5.5 Hz, 1H), 3.46 – 3.41 (m, 1H), 3.36 – 3.25 (m, 3H), 2.74 (t, J = 7.0 Hz, 2H), 2.30 (td, J = 7.5, 1.4 Hz, 2H), 2.03 (q, J = 7.2 Hz, 4H), 1.52 (q, J = 7.2 Hz, 2H), 1.34 – 1.30 (m, 4H), 1.28 – 1.22 (m, 10H), 0.87 (t, J = 7.0 Hz, 3H).

^{13}C NMR (176 MHz, DMSO-d_6): δ 173.37, 130.22, 130.20, 128.24, 128.21, 104.52, 104.41, 75.73, 73.79, 73.75, 71.07, 70.91, 70.81, 68.57, 67.91, 65.97, 65.78, 60.86, 33.94, 31.36, 29.47, 29.19, 29.05, 28.99, 28.93, 27.10, 27.08, 25.69, 24.90, 22.43, 14.38.

MS (ESI): 561.3 $[M+HCOO]^-$.

1-glycerol-β-D-galactopyranoside-(*9Z,12Z*)-9/10/12/13-octadecadienoate hydroperoxides (19-22).

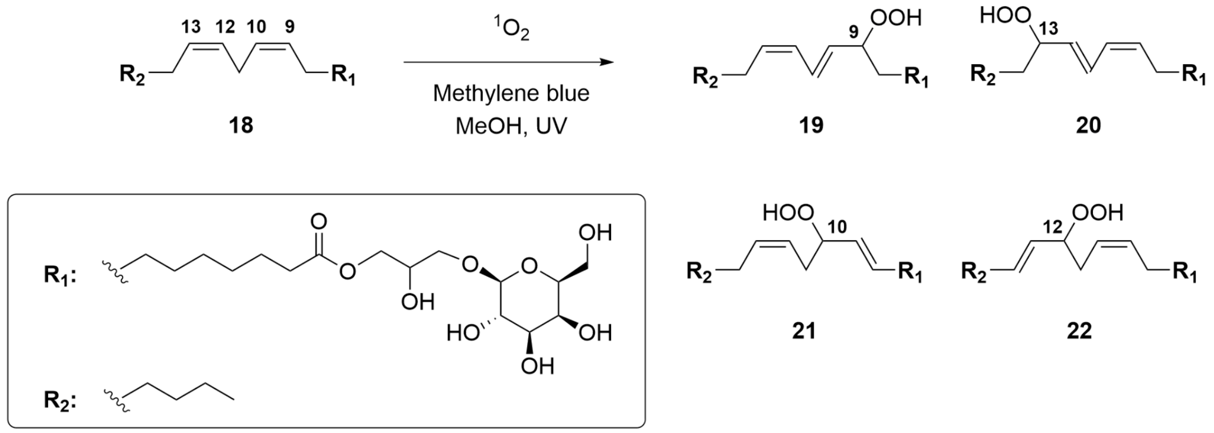

Preparation of peroxides **19-22** from 20 mg (0.04 mmol) of **18** was carried out according to methylene blue catalyzed photo-oxidation procedure described above. Mixture of isomeric hydroperoxides **19-22** was isolated using column chromatography ($CHCl_3$:MeOH 7:3): 6 mg (0.01 mmol, 28%) of the mixture of **19-22**.

^{1}H NMR (700 MHz, DMSO-d_6): δ 11.28 (d, J = 1.5 Hz, 2 x 1H), 11.26 (d, J = 1.0 Hz, 2 x 1H), 6.50 – 6.43 (m, 1H), 5.98 (t, J = 11.0 Hz, 1H), 5.60 (ddd, J = 22.2, 14.8, 7.2 Hz, 2H), 5.45 – 5.40 (m, 2H), 5.37 – 5.26 (m, 2 x 2H), 4.93 (dd, J = 10.2, 5.2 Hz, 2 x 1H), 4.82 (t, J = 4.0 Hz, 2 x 1H), 4.67 (d, J = 5.3 Hz, 2 x 1H), 4.53 (dt, J = 5.9, 3.6 Hz, 2 x 1H), 4.33 (dd, J = 4.7, 2.0 Hz, 2 x 1H), 4.14 (q, J = 6.8 Hz, 1H), 4.08 (dd, J = 7.2, 5.9 Hz, 2H), 4.06 (d, J = 4.0 Hz, 1H), 4.04 (d, J = 4.0 Hz, 1H), 3.96 (ddd, J = 17.7, 11.3, 6.4 Hz, 2H), 3.80 (d, J = 14.8 Hz, 2H), 3.69 (dt, J = 10.8, 5.6 Hz, 1H), 3.64 – 3.61 (m, 2H), 3.52 (dq, J = 12.8, 6.3 Hz, 2H), 3.49 – 3.44 (m, 2H), 3.44 – 3.40 (m, 1H), 2.29 (t, J = 7.5 Hz, 4H), 2.16 (dq, J = 14.1, 7.2 Hz, 2H), 2.05 – 1.93 (m, 3H), 1.51 (s, 4H), 1.43 – 1.35 (m, 2H), 1.33 (dt, J = 13.4, 7.0 Hz, 3H), 1.26 (t, J = 6.0 Hz, 18H), 0.89 – 0.82 (m, 2 x 3H).

MS (ESI): 593.4 $[M+HCOO]^-$.

(*9Z,11E*)-13-(2-Methoxypropan-2-ylperoxy)octadeca-9,11- dienoic acid (23).

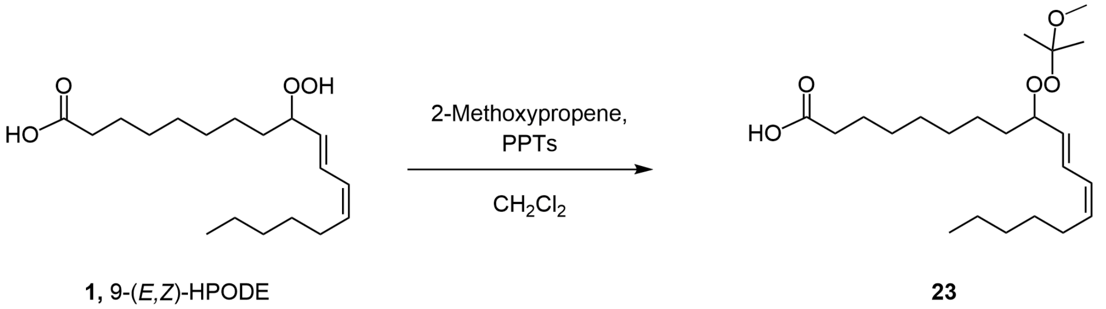

1 (78 mg, 0.25 mmol) and pyridinium p-toluene sulfonate (PPTs, 6.5 mg) were dissolved in dry CH_2Cl_2 (5 mL). Then solution of 2-methoxypropene in 1 ml CH_2Cl_2 (65 µL, excess) was added dropwise via a syringe. The resulting mixture was stirred for 2 h under argon. Then solvents were removed by rotary evaporation, and the

crude product was then flash chromatographed with hexane:ethyl acetate system 2:1 to afford pure **23** (73 mg, yield = 76%).

^{1}H NMR (300 MHz, $CDCl_3$): δ 6.48 (dd, *J* = 15.3, 11.1 Hz, 1H), 6.01 (q, *J* = 12.9, 11.0 Hz, 1H), 5.60 (dd, *J* = 15.3, 8.0 Hz, 1H), 5.45 (dt, *J* = 10.7, 7.6 Hz, 1H), 4.38 (dt, *J* = 13.9, 6.7 Hz, 1H), 3.30 (s, 3H), 2.34 (t, *J* = 7.5 Hz, 2H), 2.18 (q, *J* = 7.3 Hz, 2H), 2.07 (q, *J* = 7.1 Hz, 1H), 1.62 (d, *J* = 10.2 Hz, 2H), 1.48 (t, *J* = 7.0 Hz, 1H), 1.38 (s, 6H), 1.35 – 1.26 (m, 14H), 0.88 (t, *J* = 6.5 Hz, 3H).

^{13}C NMR (75 MHz, $CDCl_3$): δ 179.60, 133.13, 132.83, 128.18, 128.05, 104.74, 84.80, 49.40, 34.08, 33.23, 31.55, 29.41, 29.16, 29.13, 29.12, 27.86, 25.45, 24.77, 23.19, 22.97, 22.66, 14.18.

MS (ESI): 383.3 $[M-H]^-$.

(*10E,12Z*)-9-(2-Methoxypropan-2-ylperoxy)octadeca-10,12- dienoic acid (24).

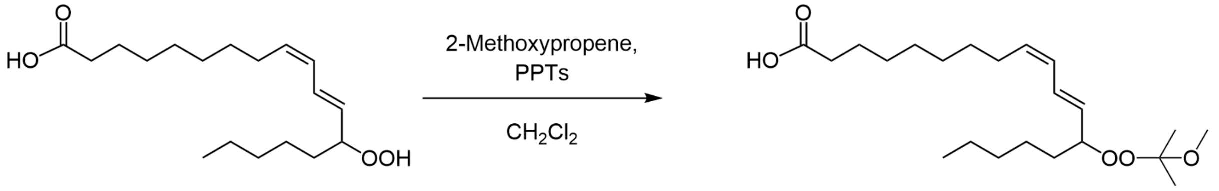

2, 13-(*Z,E*)-HPODE

24

2 (80 mg, 0.26 mmol) and pyridinium p-toluene sulfonate (PPTs, 7 mg) were dissolved in dry CH_2Cl_2 (5 mL). Then solution of 2-methoxypropene in 1 ml CH_2Cl_2 (68 µL, excess) was added dropwise via a syringe. The resulting mixture was stirred for 2 h under argon. Then solvents were removed by rotary evaporation, and the crude product was then flash chromatographed with hexane:ethyl acetate system 2:1 to afford the pure product **24** (74 mg, yield = 75%).

^{1}H NMR (300 MHz, $CDCl_3$): δ 6.47 (dd, *J* = 15.3, 11.1 Hz, 1H), 5.99 (t, *J* = 10.9 Hz, 1H), 5.60 (dd, *J* = 15.0, 7.9 Hz, 1H), 5.45 (dt, *J* = 10.7, 7.6 Hz, 1H), 4.40 (dd, *J* = 13.2, 5.6 Hz, 2H), 3.30 (s, 3H), 2.35 (t, *J* = 7.6 Hz, 2H), 2.22 – 2.13 (m, 2H), 1.63 (d, *J* = 10.2 Hz, 2H), 1.38 (s, 6H), 1.36-1.21 (m, 14H), 0.86 (t, *J* = 6.5 Hz, 3H).

MS (ESI): 383.3 $[M-H]^-$.

1-glycerol-(2', 3', 4', 6'-Tetra-O-acetyl-β-D-galactopyranosyl)-(*10E,12Z*)-9-(2-Methoxypropan-2-ylperoxy)octadeca-10,12-dienoat (25).

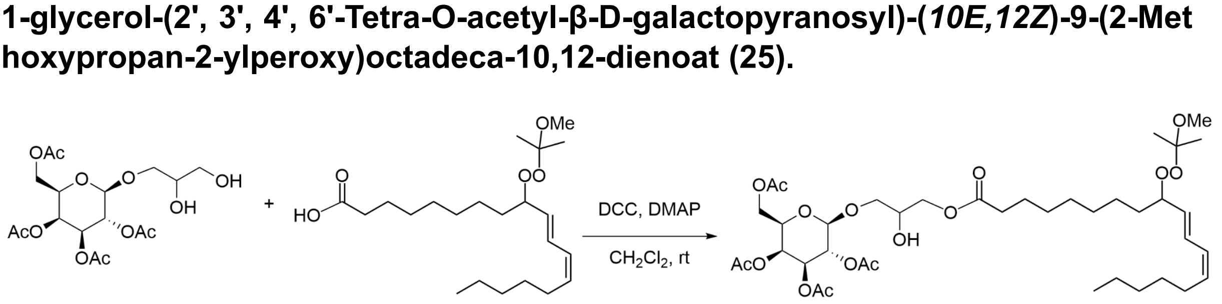

16 **23** **25**

DCC (10 mg, 0.05 mmol) and DMAP (0.5 mg, 0.004 mmol) were added to a solution of the **23** (17 mg, 0.044 mmol) in 1 ml DCM, and the mixture was stirred for half an hour in an inert atmosphere. Then, solution of **16** (21 mg, 0,05 mmol) in 1 ml DCM

was added dropwise to the mixture and left stirred overnight at room temperature. The precipitate that formed was filtered off, the solvent was evaporated, and the product **25** was isolated using column chromatography Hex:EtOAc 2:1 → Hex:EtOAc 1:1: colorless oily liquid (19 mg, 56%).

^{1}H NMR (300 MHz, $CDCl_3$): δ 6.48 (dd, *J* = 15.3, 11.1 Hz, 1H), 5.99 (t, *J* = 11.0 Hz, 1H), 5.59 (dd, *J* = 15.3, 8.0 Hz, 1H), 5.52 – 5.40 (m, 1H), 5.39 (dd, *J* = 3.5, 1.1 Hz, 1H), 5.21 (ddd, *J* = 10.5, 7.9, 1.3 Hz, 1H), 5.02 (dd, *J* = 10.5, 3.4 Hz, 1H), 4.50 (dd, *J* = 7.9, 4.4 Hz, 1H), 4.38 (p, *J* = 6.4 Hz, 1H), 4.18 – 4.09 (m, 4H), 4.01 (q, *J* = 4.8 Hz, 1H), 3.97 – 3.90 (m, 1H), 3.81 – 3.75 (m, 1H), 3.68 (dd, *J* = 10.5, 5.9 Hz, 1H), 3.29 (d, *J* = 1.4 Hz, 3H), 2.84 (d, *J* = 4.1 Hz, 1H), 2.69 (d, *J* = 5.8 Hz, 1H), 2.33 (t, *J* = 7.5 Hz, 2H), 2.16, 2.07, 2.06, 1.99 (all s, 12H, 4×C(O)CH_3), 1.38 (s, 6H), 1.33 – 1.24 (m, 19H), 0.91 – 0.85 (m, 3H).

MS (ESI): 833.4 [M+HCOO]$^-$.

1-glycerol-β-D-galactopyranosyl-(*10E,12Z*)-9-(2-methoxypropan-2-ylperoxy)octadeca-10,12-dienoate (26).

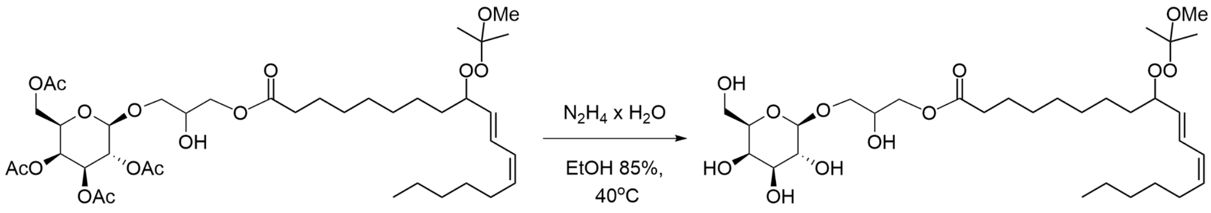

25 **26**

To a solution of **25** (19 mg, 0.02 mmol) in 1 ml of 85% ethanol was added hydrazine monohydrate (12 mg, 0.23 mmol). The resulting mixture was stirred at 40°C until TLC ($CHCl_3$:EtOH, 4:1) showed complete conversion to a more polar product (Rf = 0.32), then the solution was poured into ice water (3 ml) and extracted with chloroform (3 x 2 ml). The combined organic phase was dried over anhydrous Na_2SO_4, the solvent was evaporated, product **26** was used in the next step without further purification.

MS (ESI): 665.6 [M+HCOO]$^-$.

1-glycerol-β-D-galactopyranosyl-(*10E,12Z*)-9-hydroperoxyoctadeca-10,12-dienoate (27).

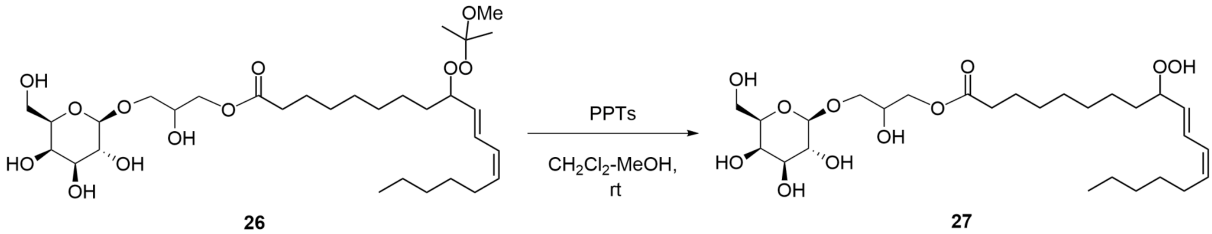

26 **27**

26 (6 mg, 0.01 mmol) and pyridinium p-toluene sulfonate (PPTs, 0.4 mg, 0.002 mmol) were dissolved into 1 mL of CH_2Cl_2/methanol (1/1) and the solution stirred under argon until TLC ($CHCl_3$:EtOH, 4:1) showed complete conversion to a more polar product (Rf = 0.26). The solvents were then removed by rotary evaporation. The residue was dissolved into water (3 mL) and extracted with chloroform (3 x 1 mL) to

remove the PPTs. The combined organic phase was dried over anhydrous Na_2SO_4, the solvent was evaporated, the product **27** was isolated using column chromatography ($CHCl_3$:EtOH 9:1 → $CHCl_3$:EtOH 7:3): colorless oily liquid (3 mg, 63%).

MS (ESI): 593.4 [M+HCOO]$^-$.

1-glycerol-(2', 3', 4', 6'-Tetra-O-acetyl-β-D-galactopyranosyl)-(*9Z,11E*)-13-(2-Methoxypropan-2-ylperoxy)octadeca-9,11-dienoate (28).

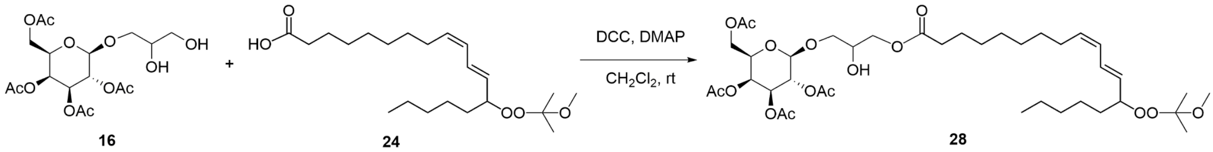

DCC (148 mg, 0.72 mmol) and DMAP (8 mg, 0.0065 mmol) were added to a solution of the **24** (250 mg, 0.65 mmol) in 4 ml DCM, and the mixture was stirred for half an hour in an inert atmosphere. Then, a solution of **16** (275 mg, 0,65 mmol) in 2 ml DCM was added dropwise to the mixture and left stirred overnight at room temperature. The precipitate that formed was filtered off, the solvent was evaporated, and the product **28** was isolated using column chromatography Hex:EtOAc 2:1 → Hex:EtOAc 1:1: colorless oily liquid (163 mg, 66%).

^{1}H NMR (300 MHz, $CDCl_3$): δ 6.48 (dd, *J* = 15.2, 11.1 Hz, 1H), 5.98 (t, *J* = 10.9 Hz, 1H), 5.60 (dd, *J* = 15.2, 7.9 Hz, 1H), 5.50 – 5.42 (m, 1H), 5.39 (dd, *J* = 3.5, 1.1 Hz, 1H), 5.20 (ddd, *J* = 9.4, 7.9, 1.3 Hz, 1H), 5.02 (dd, *J* = 10.5, 3.4 Hz, 1H), 4.50 (dd, *J* = 7.9, 4.4 Hz, 1H), 4.38 (p, *J* = 6.3 Hz, 1H), 4.17 – 4.08 (m, 4H), 4.04 – 3.86 (m, 2H), 3.80 – 3.74 (m, 1H), 3.67 (dd, *J* = 10.6, 5.9 Hz, 1H), 3.29 (s, 3H), 2.86 (d, *J* = 4.1 Hz, 1H), 2.71 (d, *J* = 5.8 Hz, 1H), 2.32 (t, *J* = 7.6 Hz, 2H), 2.17, 2.16, 2.07, 2.05, 1.98 (all s, 12H, 4×C(O)CH_3), 1.37 (s, 6H), 1.34 – 1.24 (m, 19H), 0.93 – 0.83 (m, 3H).

MS (ESI): 833.4 [M+HCOO]$^-$.

1-glycerol-β-D-galactopyranosyl-(*9Z,11E*)-13-(2-Methoxypropan-2-ylperoxy)octadeca-9,11-dienoate (29).

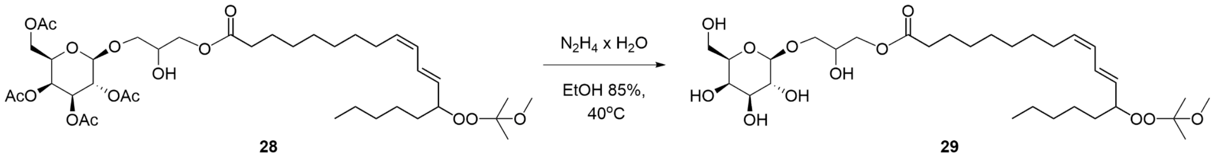

To a solution of **28** (118 mg, 0.15 mmol) in 3 ml of 85% ethanol was added hydrazine monohydrate (70 mg, 1.4 mmol). The resulting mixture was stirred at 40°C until TLC ($CHCl_3$:EtOH, 4:1) showed complete conversion to a more polar product (Rf = 0.33), then the solution was poured into ice water (10 ml) and extracted with chloroform (3 x 5 ml). The combined organic phase was dried over anhydrous Na_2SO_4, the solvent was evaporated, product **29** was isolated using column chromatography ($CHCl_3$:EtOH 9:1 → $CHCl_3$:EtOH 7:3): colorless oily liquid (33 mg, 35%).

^{1}**H NMR** (300 MHz, $CDCl_3$): δ 6.47 (dd, *J* = 15.3, 11.1 Hz, 1H), 5.98 (t, *J* = 11.0 Hz, 1H), 5.60 (dd, *J* = 15.3, 7.9 Hz, 1H), 5.48 – 5.38 (m, 1H), 5.37 – 5.29 (m, 1H), 4.40 (p, *J* = 6.3 Hz, 1H), 4.31 (d, *J* = 6.2 Hz, 1H), 4.11 (s, 2H), 4.09 – 3.99 (m, 1H), 3.87 – 3.74 (m, 2H), 3.71 – 3.51 (m, 3H), 3.29 (s, 3H), 2.33 (t, *J* = 7.6 Hz, 2H), 2.22 – 2.12 (m, 2H), 2.09 – 2.01 (m, 1H), 1.72 – 1.53 (m, 5H), 1.38 (s, 6H), 1.34 – 1.20 (m, 21H), 0.88 (t, *J* = 6.4 Hz, 3H).

MS (ESI): 665.6 $[M+HCOO]^-$.

1-glycerol-β-D-galactopyranosyl-(*9Z,11E*)-13-hydroperoxyoctadeca-9,11-dienoate (30).

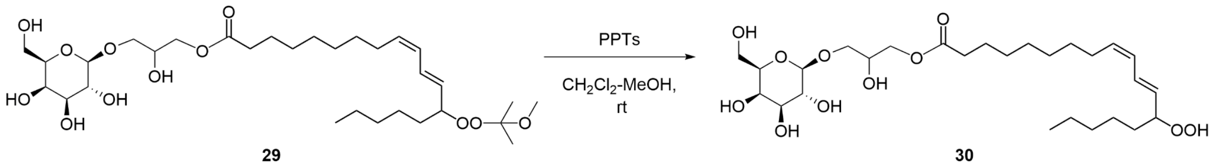

29 (33 mg, 0.05 mmol) and pyridinium p-toluene sulfonate (PPTs, 2 mg, 0.009 mmol) were dissolved into 5 mL of CH_2Cl_2/methanol (1/1) and the solution stirred under argon until TLC ($CHCl_3$:EtOH, 4:1) showed complete conversion to a more polar product (Rf = 0.26). The solvents were then removed by rotary evaporation. The residue was dissolved into water (10 mL) and extracted with chloroform (3 x 5 mL) to remove the PPTs. The combined organic phase was dried over anhydrous Na_2SO_4, the solvent was evaporated, product **30** was isolated using column chromatography ($CHCl_3$:EtOH 9:1 → $CHCl_3$:EtOH 7:3): colorless oily liquid (22 mg, 78%).

^{1}**H NMR** (700 MHz, DMSO-d_6): δ 11.26 (s, 1H), 6.46 (dd, *J* = 15.3, 11.1 Hz, 1H), 5.98 (t, *J* = 10.9 Hz, 1H), 5.59 (dd, *J* = 15.3, 7.7 Hz, 1H), 5.42 (dt, *J* = 10.6, 7.6 Hz, 1H), 4.93 (d, *J* = 4.8 Hz, 1H), 4.92 (d, *J* = 5.8 Hz, 2H), 4.81 (d, *J* = 4.1 Hz, 3H), 4.66 (d, *J* = 5.4 Hz, 3H), 4.53 – 4.51 (m, 3H), 4.32 (d, *J* = 2.8 Hz, 2H), 4.08 (d, *J* = 6.8 Hz, 3H), 4.05 (dd, *J* = 11.6, 3.9 Hz, 3H), 3.99 – 3.96 (m, 1H), 3.81 (q, *J* = 5.1 Hz, 4H), 3.69 (dt, *J* = 11.0, 5.6 Hz, 4H), 3.62 (t, *J* = 3.5 Hz, 5H), 3.53 (dt, *J* = 11.8, 6.1 Hz, 4H), 3.47 (q, *J* = 5.6 Hz, 3H), 3.44 – 3.41 (m, 3H), 3.34 – 3.32 (m, 3H), 3.28 (d, *J* = 3.7 Hz, 0H), 3.26 (d, *J* = 3.8 Hz, 0H), 2.28 (d, *J* = 7.6 Hz, 9H), 2.15 (q, *J* = 7.1 Hz, 2H), 1.34 (q, *J* = 6.8, 6.3 Hz, 1H), 1.26 (s, 27H), 0.88 – 0.83 (m, 3H).

MS (ESI): 593.4 $[M+HCOO]^-$.

1-palmitoyl-2-(*9Z,11E*)-13-hydroperoxyoctadeca-9,11-dienoat-sn-glycero-3-phosphocholine (31).

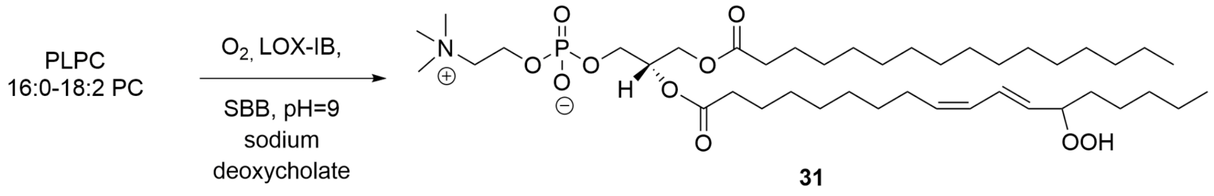

Solution of PLPC (10 mg/0.5 ml of ethanol) was mixed with 10 ml of 50 mM sodium borate buffer (SBB, pH=9.0) containing 2 mg of soybean lipoxygenase-1 (LOX-1B,

1.25 x 10^6 units; Sigma Aldrich) and sodium deoxycholate (50 mg). Oxygen was bubbled through the resulting mixture. The progress of the reaction was monitored by TLC ($CHCl_3$:MeOH:H_2O 70:27:3). After 16 hours the conversion stopped changing, 1M HCl was added to the reaction mixture to pH=4 and the product **31** was extracted with diethyl ether (3 x 5 ml). The combined organic phase was dried over anhydrous Na_2SO_4, the solvent was evaporated. Crude product **31** was further purified using RP-LC on PuriFlash 5.250 system with UV-detector (Interchim, France) with PFC18HP-F0004 column (Interchim, France) eluted with/water linear gradient – 50-100% acetonitrile, flow rate = 10 mL/min, λ = 235 nm.

^{1}H NMR (700 MHz, CD_3OD:$CDCl_3$:D_2O 16:9:1): δ 6.63 (dd, *J* = 15.3, 11.1 Hz, 1H), 6.11 (t, *J* = 10.9 Hz, 1H), 5.69 (dd, *J* = 15.3, 8.1 Hz, 1H), 5.56 (dt, *J* = 10.9, 7.6 Hz, 1H), 5.33 (dtd, *J* = 10.6, 5.6, 3.0 Hz, 1H), 4.51 (dd, *J* = 12.1, 3.1 Hz, 1H), 4.43 (q, *J* = 7.0 Hz, 1H), 4.36 (d, *J* = 8.9 Hz, 2H), 4.26 (dd, *J* = 12.1, 7.2 Hz, 1H), 4.09 (dd, *J* = 6.9, 5.5 Hz, 2H), 3.72 – 3.70 (m, 2H), 3.44 (p, *J* = 1.6 Hz, 4H), 2.46 – 2.39 (m, 4H), 2.30 (tddd, *J* = 7.4, 5.0, 3.7, 1.5 Hz, 2H), 1.77 (ddt, *J* = 13.4, 9.9, 6.1 Hz, 2H), 1.70 (q, *J* = 7.7 Hz, 6H), 1.59 – 1.53 (m, 2H), 1.51 – 1.47 (m, 2H), 1.45 – 1.34 (m, 38H), 0.99 (td, *J* = 7.1, 1.8 Hz, 6H).

^{13}C NMR (176 MHz, CD_3OD:$CDCl_3$:D_2O 16:9:1): δ 174.14, 173.74, 133.03, 132.12, 128.92, 127.80, 86.37, 70.44, 70.40, 66.37, 63.58, 63.56, 62.70, 59.09, 59.06, 54.07, 54.05, 54.03, 34.11, 34.01, 32.58, 31.82, 31.69, 29.58, 29.56, 29.54, 29.41, 29.40, 29.24, 29.21, 29.05, 29.01, 28.91, 28.90, 27.59, 24.86, 24.77, 24.74, 22.56, 22.40, 13.84, 13.73.

NMR data of synthetic compounds

Table S4. Chemical shifts and relevant NMR data for the compound **13-HPODE** (d_6-DMSO, 30°C, 700MHz).

Position	^{13}C	^{1}H	multiplicity	HMBC/TOCSY
**1**	174.94	-		
**1-OH**	-	11.89	br.[a]	-
**2**	34.15	2.185	t(7)[c]	1,3,4/3,4[b]
**3**	24.97	1.487	p(7)	1,2,4/2,4
**4**	29.016	1.261	ov.	-/2,3
**5**	28.895	1.270	ov.	-/7
**6**	29.022	1.273	ov.	-/7
**7**	29.47	1.341	ov.	5,6,8,9/5,6,8,9
**8**	27.54	2.154	q(7)	6,7,9,10/6,7,9,10
**9**	132.67	5.431	dt(11,8)	7,8,11/7,8,10,11
**10**	128.63	5.988	td(11,2)	8,11,12/8,9,11,12
**11**	128.02	6.469	dd(15,11)	9,10,12,13/9,10,12,13
**12**	133.78	5.598	dd(11,11)	10,11,13,14/10,11,13,14
**13**	85.14	4.240	q(7)	11,12,14,15/11,12,14,15
**13-OOH**	-	11.31	br.	-
**14**	32.78	1.593, 1.389	m/m	12,13,15,16/12,13,15,16
**15**	24.89	1.263	p(7)	13,14,16,17/13,14,16,17
**16**	31.68	1.258	ov.	18/18
**17**	22.48	1.268	ov.	18/18
**18**	14.32	0.861	t(7)	16,17/16,17

[a]ov. - overlapped signals, br. - broad signal
[b]correlations between the current proton and carbons in HMBC/HSQC-TOCSY (mixing time=10 ms) spectra
[c]multiplicity of the signal (s - singlet, d - doublet, t - triplet, q - quadruplet, p - quintet, m - complex multiplet)

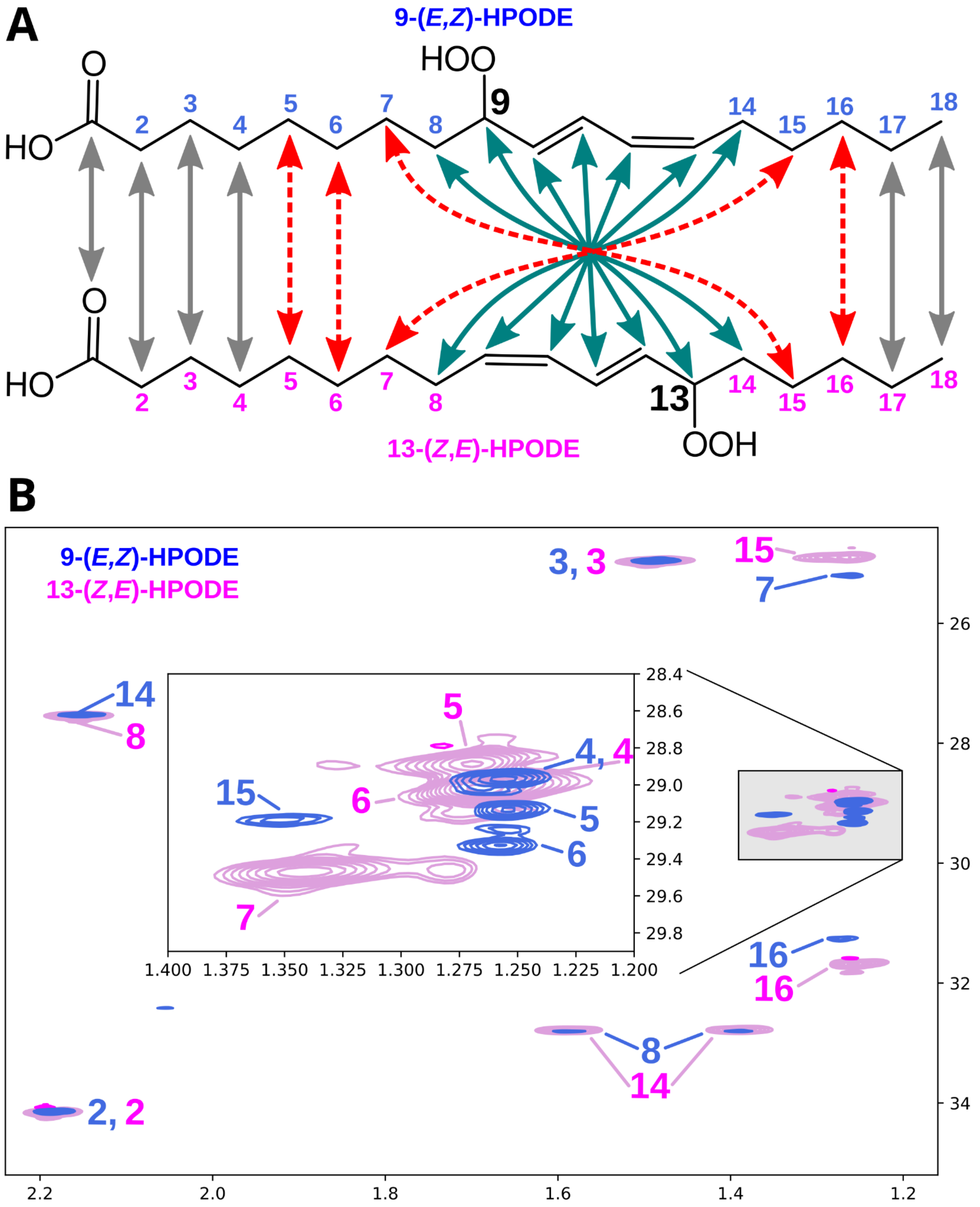

Figure S2. Comparison of NMR data of 9-(*E,Z*)-HPODE and 13-(*Z,E*)-HPODE (synthetic compounds) shows only tiny differences in ^{13}C chemical shifts of several atoms. **A**. Chemical structures with numbers. Gray arrows show full match of both ^{1}H and ^{13}C chemical shifts to the atoms of the same number, dark blue arrows outline full match in reverse order (fragment 8-14 matches to 14-8) and red dotted arrows show partial match with visible difference of either ^{1}H or ^{13}C chemical shifts. **B**. Overlay of -CH$_2$- region in ^{1}H-^{13}C HSQC with multiplicity editing of compounds 9-(*E,Z*)-HPODE (blue) and 13-(*Z,E*)-HPODE (pink), atom numbers and colours coincide with figure **A**. Conditions: d$_6$-DMSO, 30°C, 700MHz.

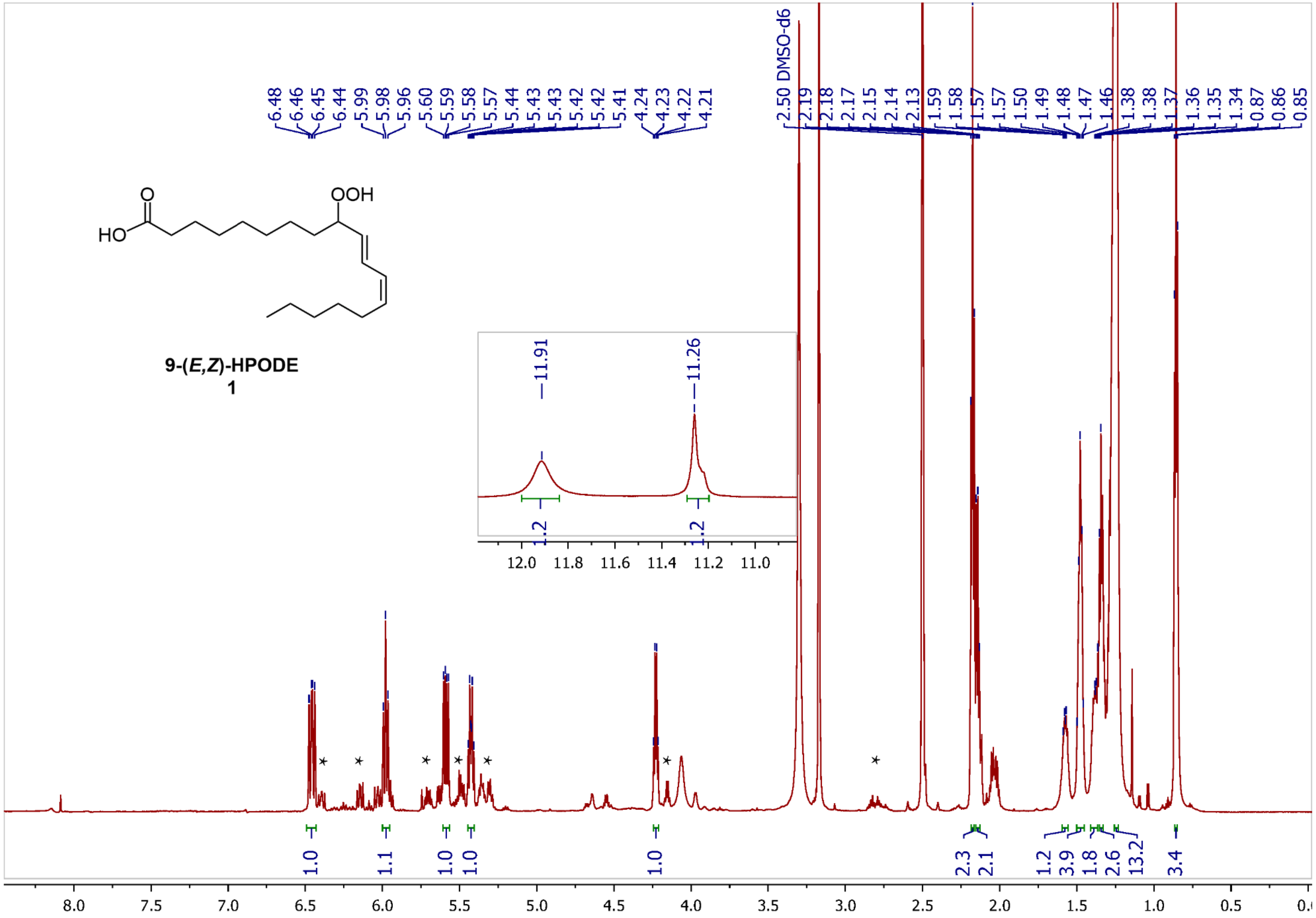

Figure S3. ^{1}H-NMR spectrum (700 MHz) of compound **1** 9-(*E*,*Z*)-HPODE in DMSO-d_6. * - 9- and 13- (*E*,*E*) isomers impurities.

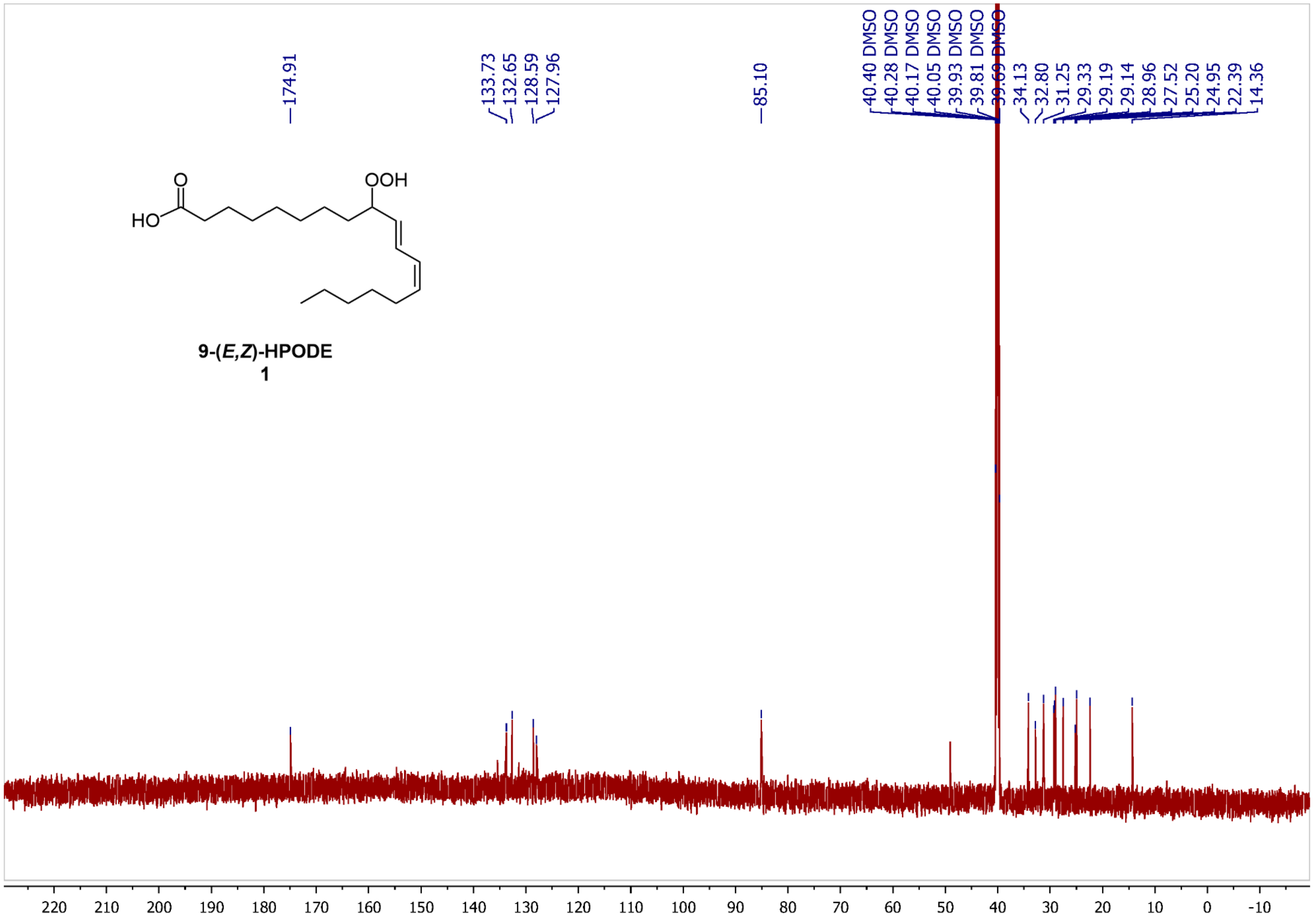

Figure S4. ^{13}C-NMR spectrum (176 MHz) of compound **1** 9-(*E,Z*)-HPODE in DMSO-d_6.

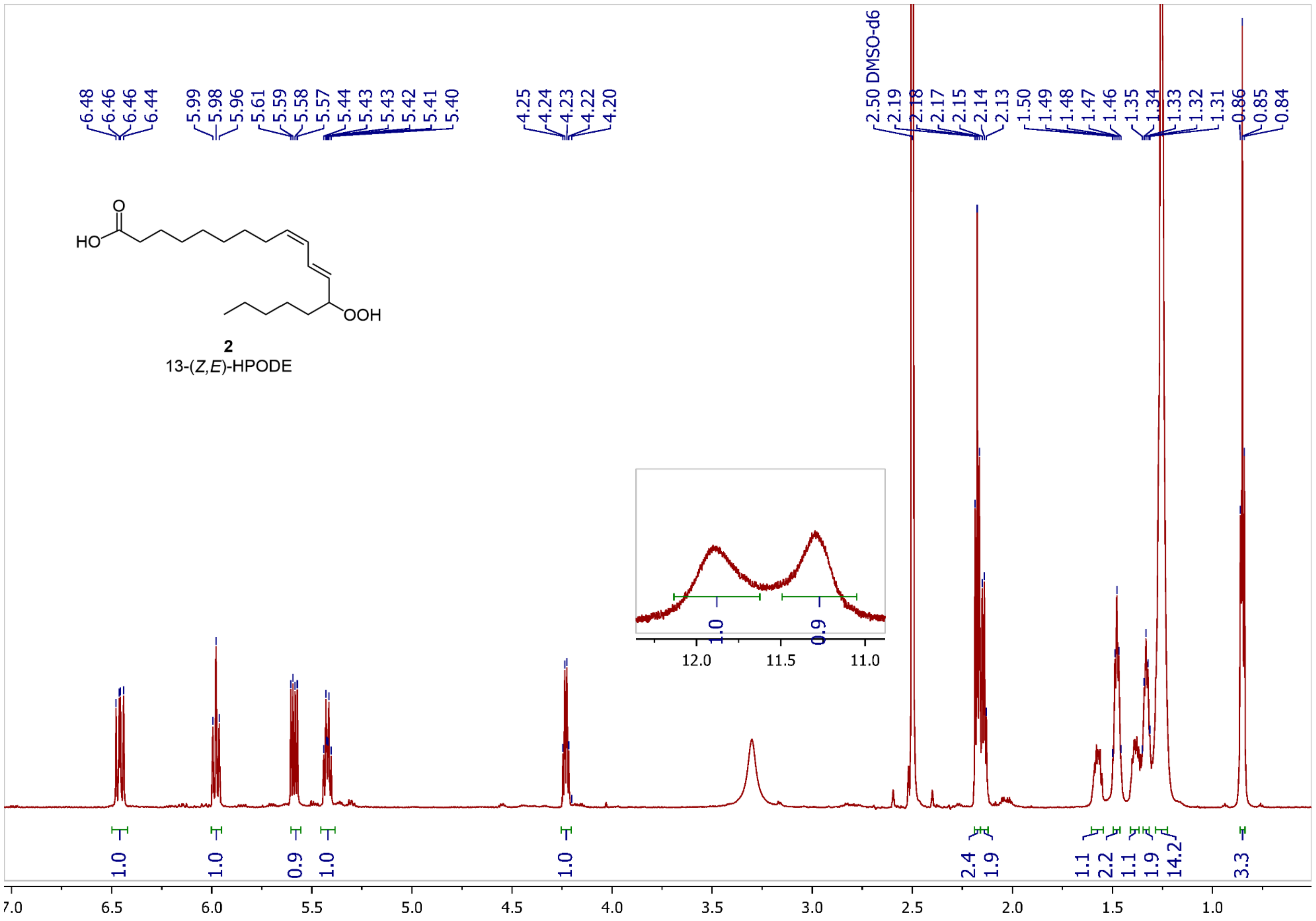

Figure S5. ^{1}H-NMR spectrum (700 MHz) of compound **2** 13-(*Z*,*E*)-HPODE in DMSO-d_6.

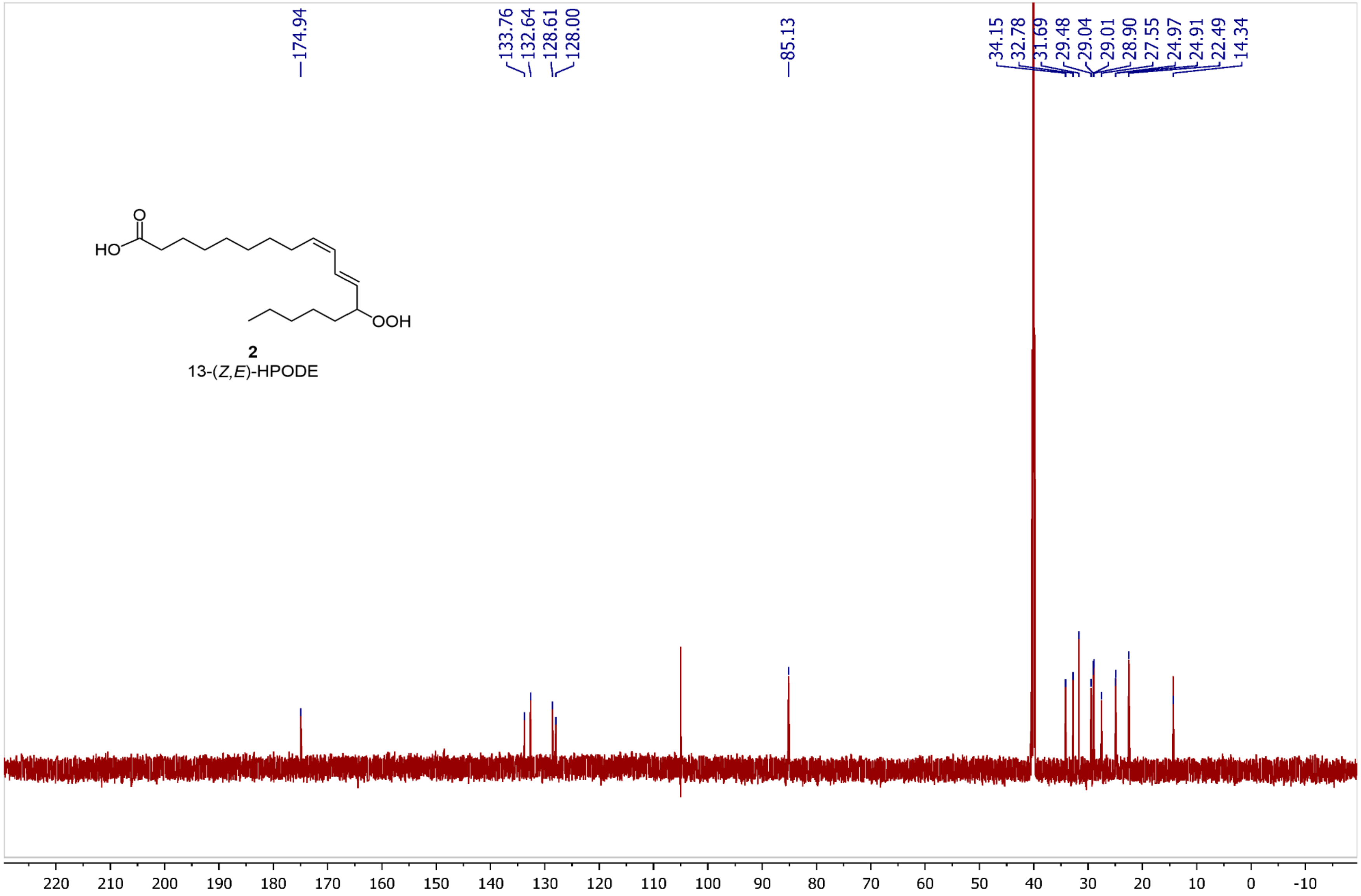

Figure S6. ^{13}C-NMR spectrum (176 MHz) of compound **2** 13-(*Z,E*)-HPODE in DMSO-d_6.

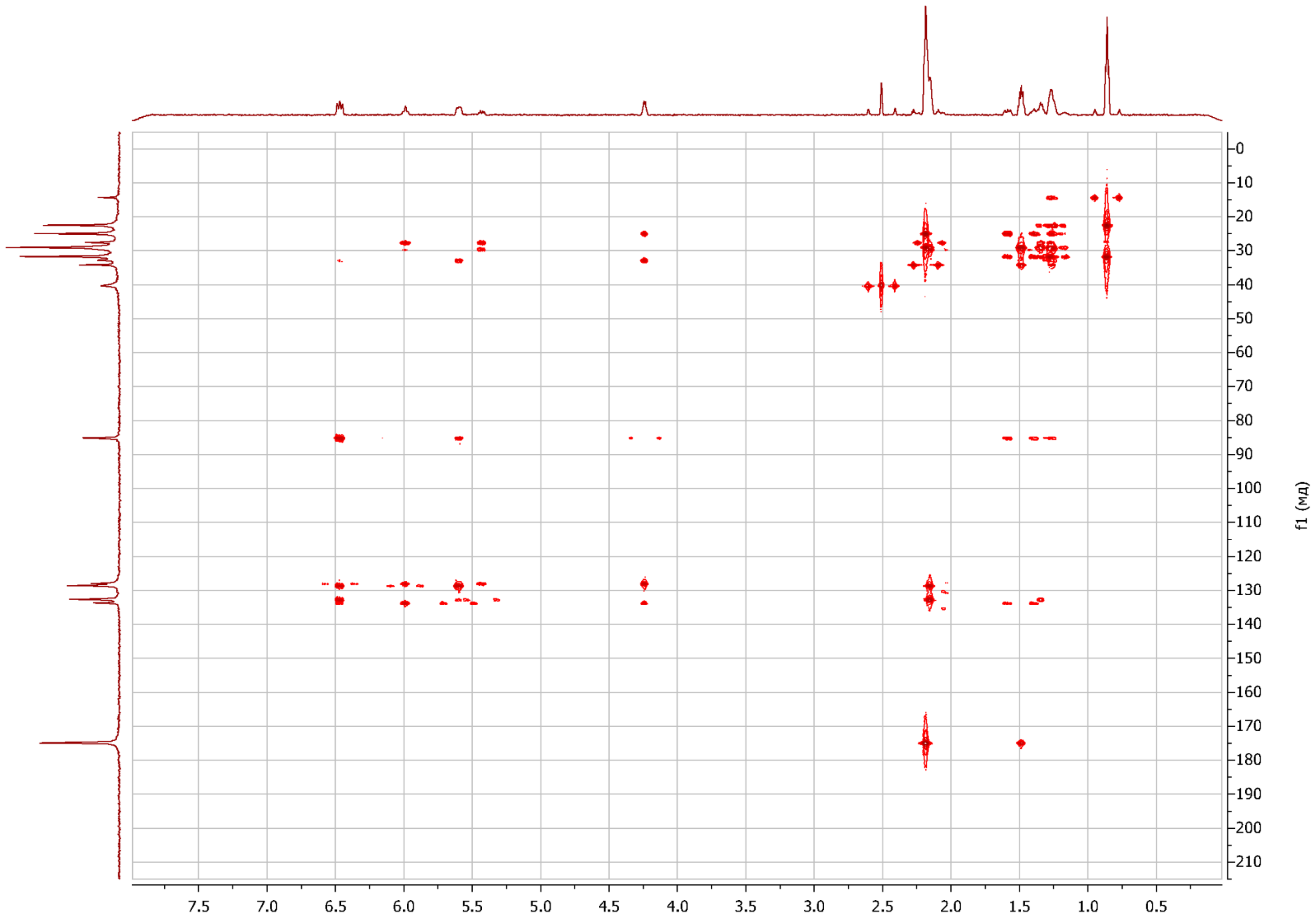

Figure S7. HSQC-NMR spectrum (700 MHz) of compound **2** 13-(*Z*,*E*)-HPODE in DMSO-d_6.

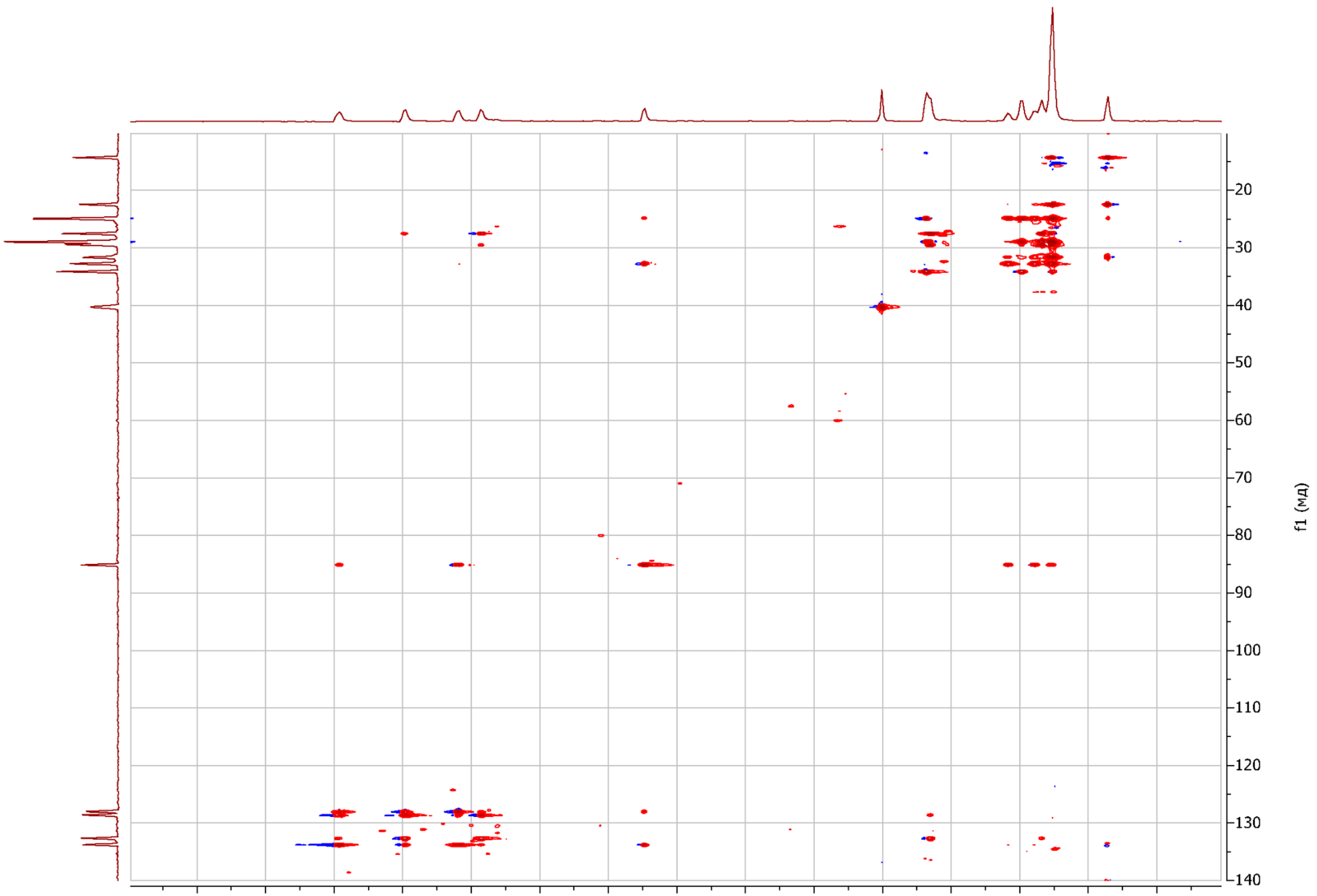

Figure S8. HMBC-NMR spectrum (700 MHz) of compound **2** 13-(*Z*,*E*)-HPODE in DMSO-d_6.

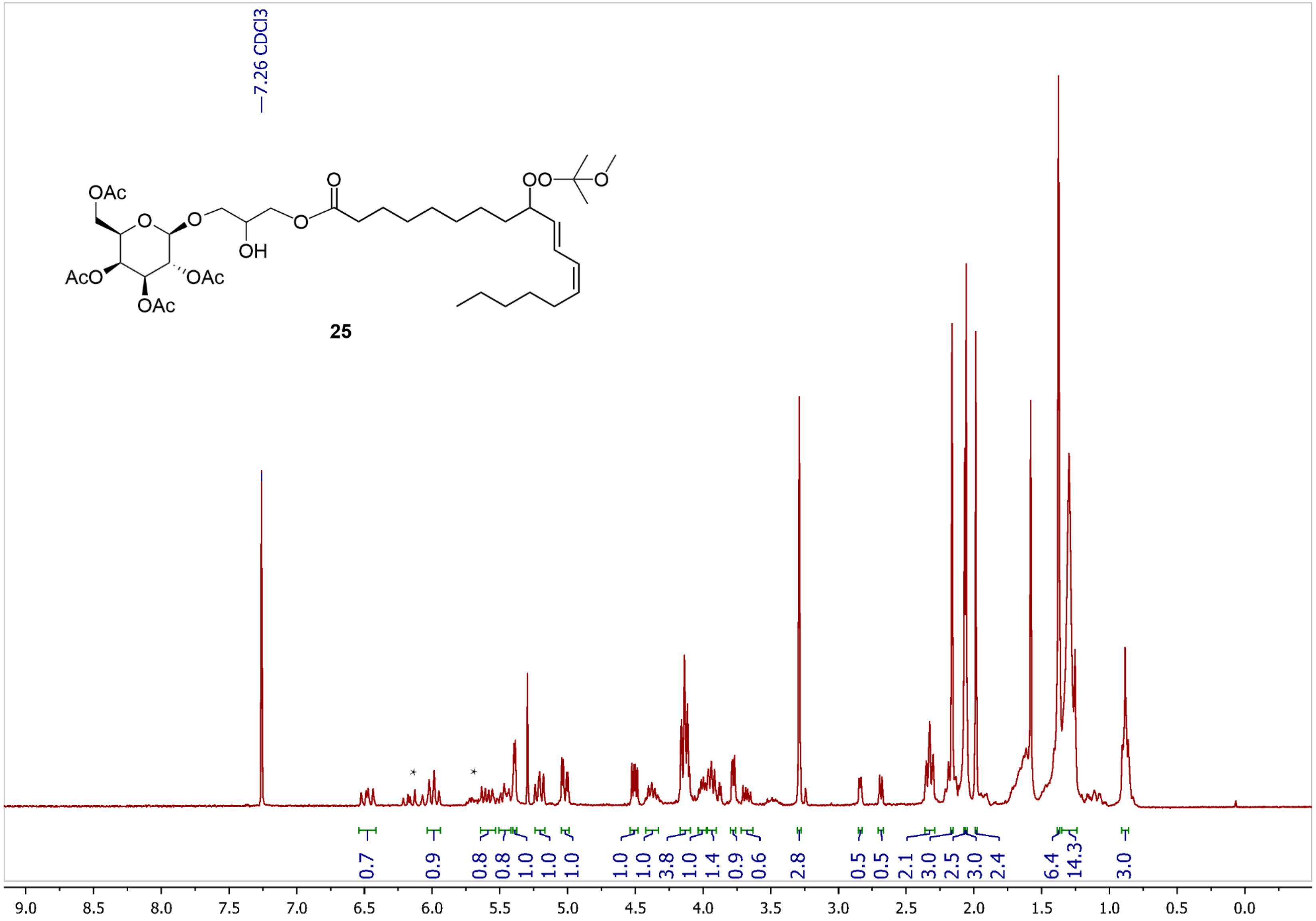

Figure S9. ^{1}H-NMR spectrum (700 MHz) of compound **25** Gal_gly_9-(*E,Z*)-HPODE in $CDCl_3$. * - 9- and 13- (*E,E*) isomers impurities.

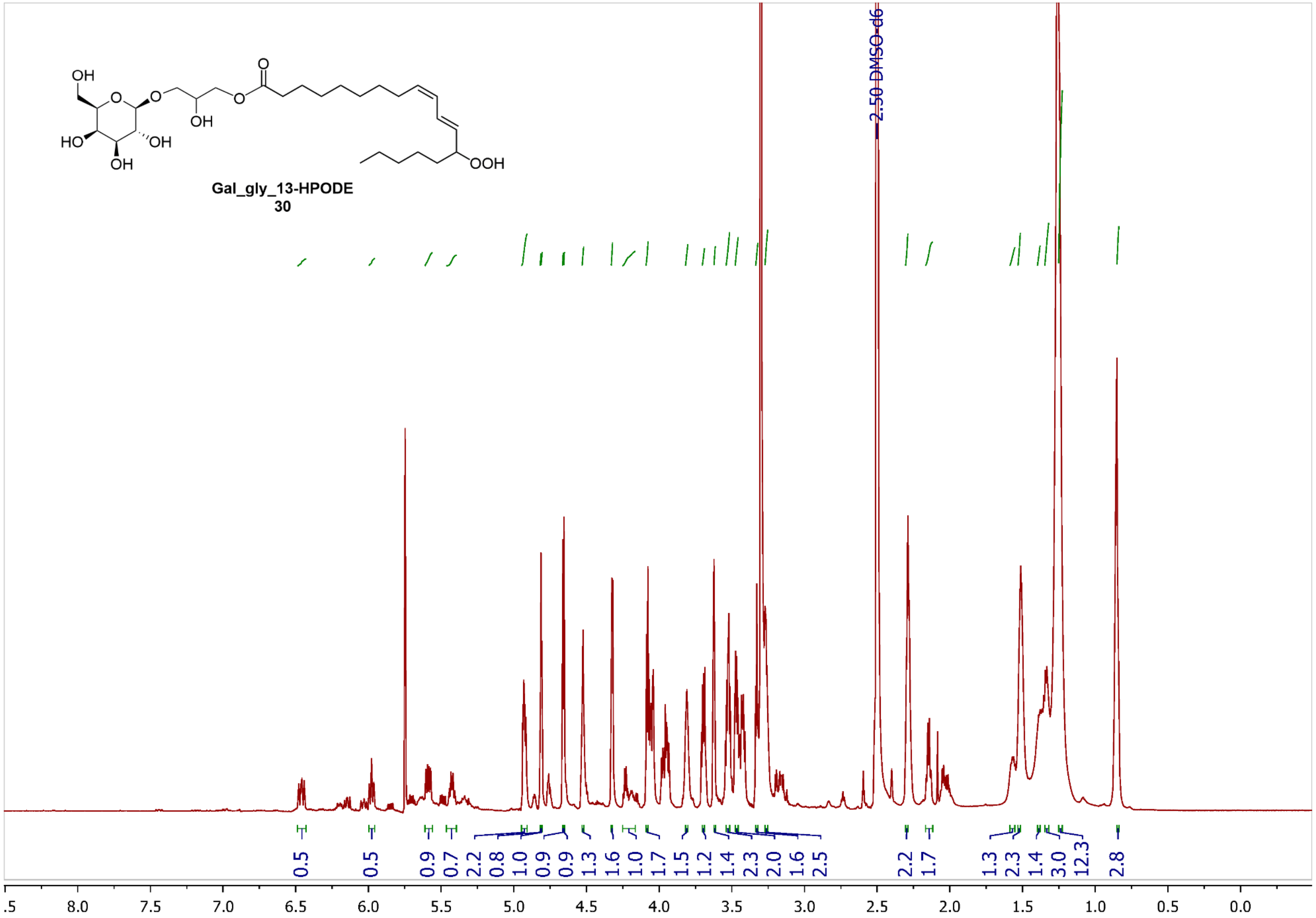

Figure S10. ^{1}H-NMR spectrum (700 MHz) of compound **30** Gal_gly_13-(*Z,E*)-HPODE in DMSO-d_6.

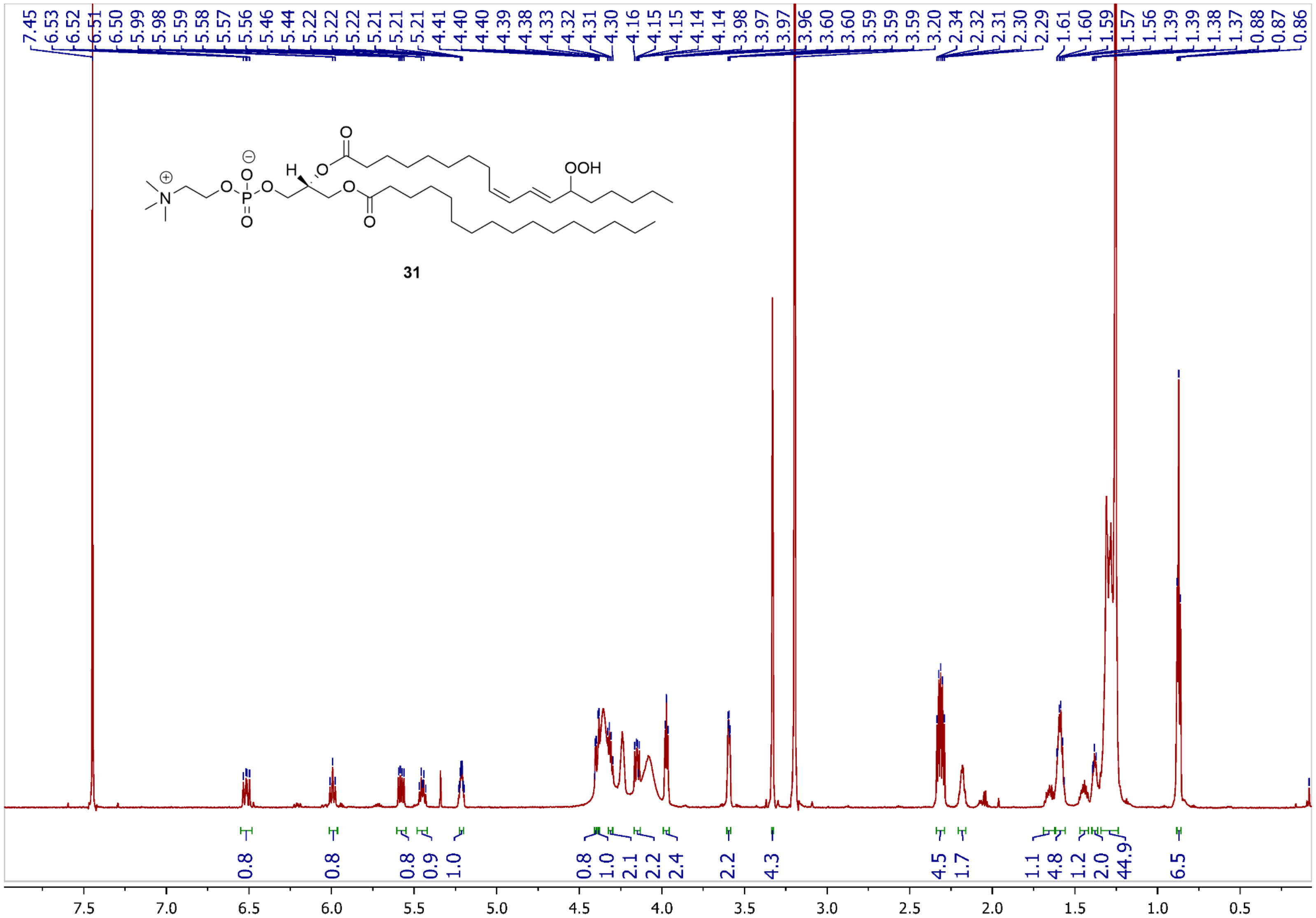

Figure S11. ^{1}H-NMR spectrum (700 MHz) of compound **31** in $CD_3OD:CDCl_3:D_2O$ 16:9:1.

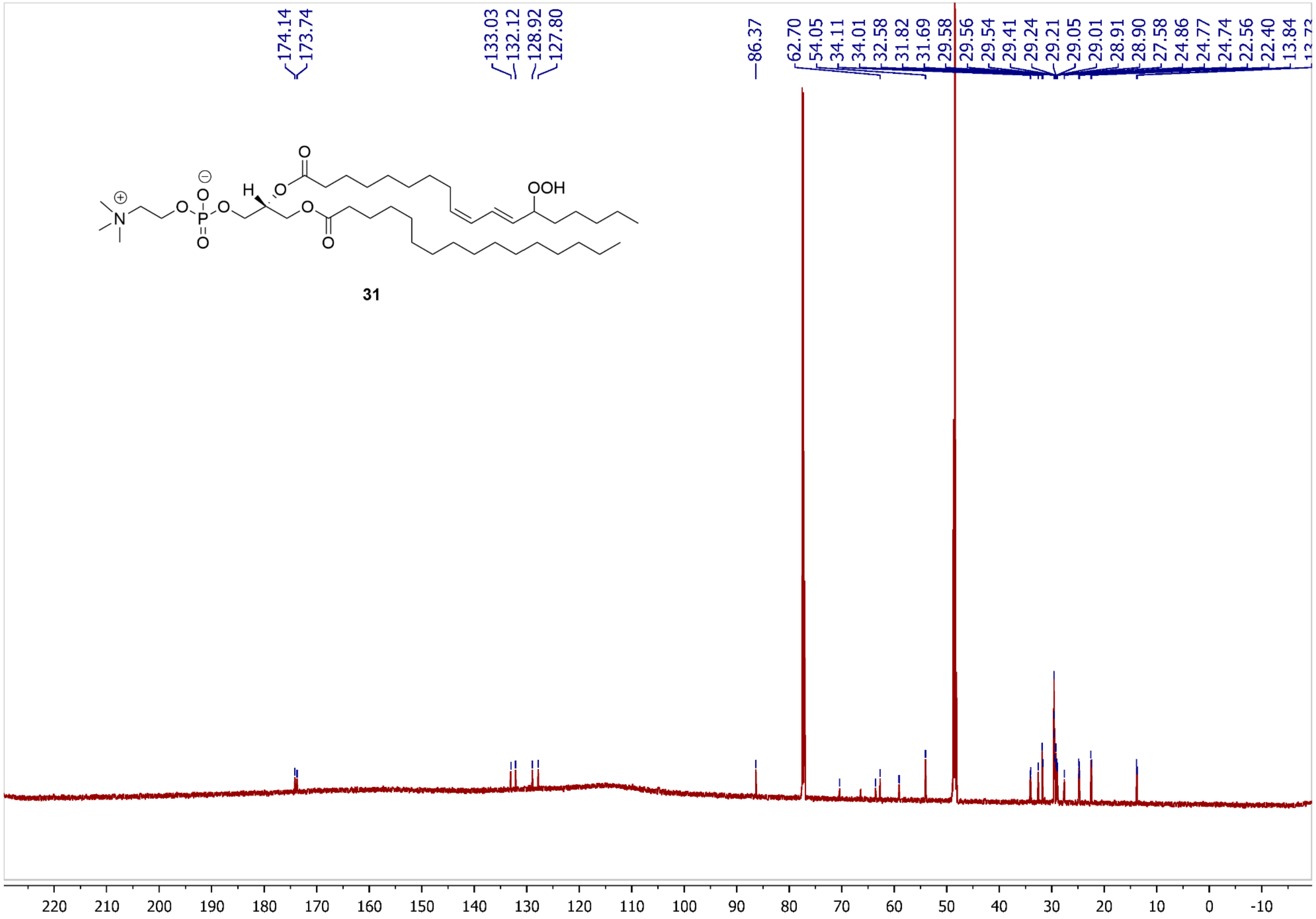

Figure S12. ^{13}C-NMR spectrum (176 MHz) of compound **31** in CD_3OD:$CDCl_3$:D_2O 16:9:1.

Bioluminescence activity assay

Reactions were monitored with a custom made luminometer BLM (Oberon-K, Krasnoyarsk, Russia). For each measurement 100 µl of reaction mixture (phosphate-buffered saline (pH 7.4)), 1 µl of luciferase fraction, 1 ul of 3mM peroxide methanolic solution, 1 ul 10 mM $FeSO_4$ solution) were used. Measurements were corrected for background luminescence of polyunsaturated fatty acids peroxides in the presence of Fe^{2+} ions. Since the substrates have slightly different kinetics, activity data are given as the area under the curve of the kinetics of the luminescent reaction.

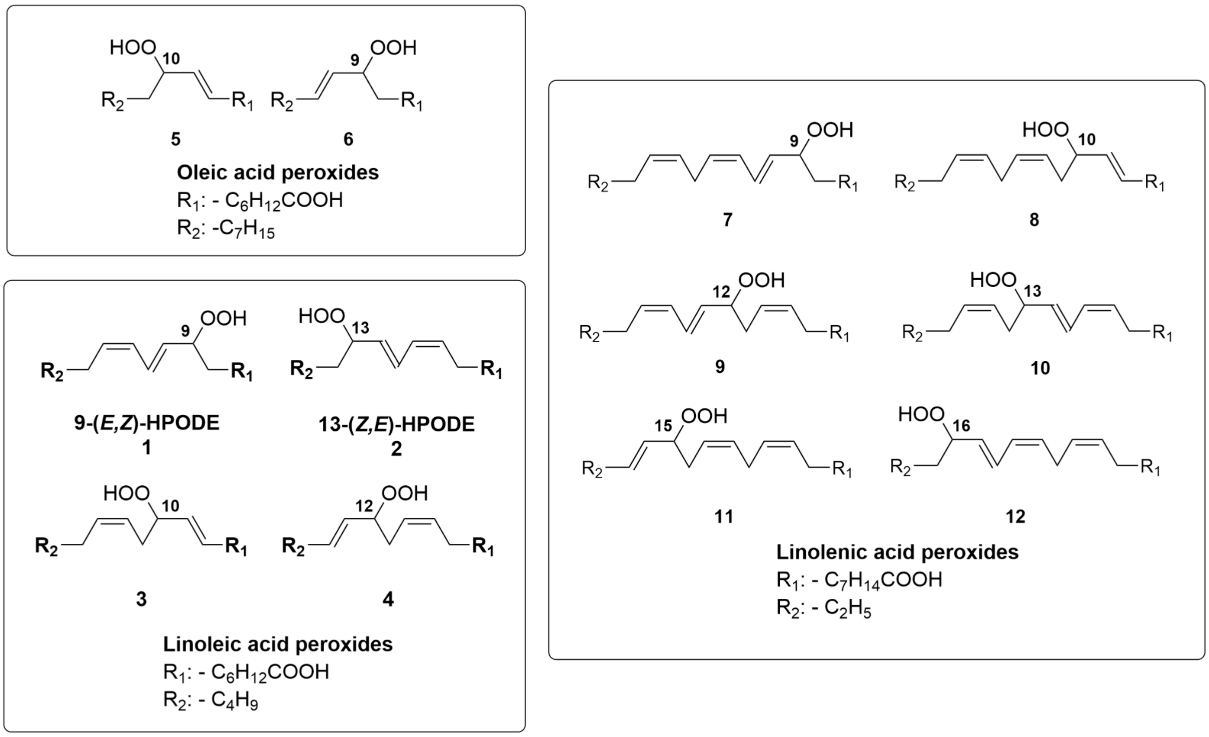

Figure S13. Mixtures of peroxides of oleic, linoleic and linolenic acids obtained by oxidation with singlet oxygen used in bioluminescent activity test.

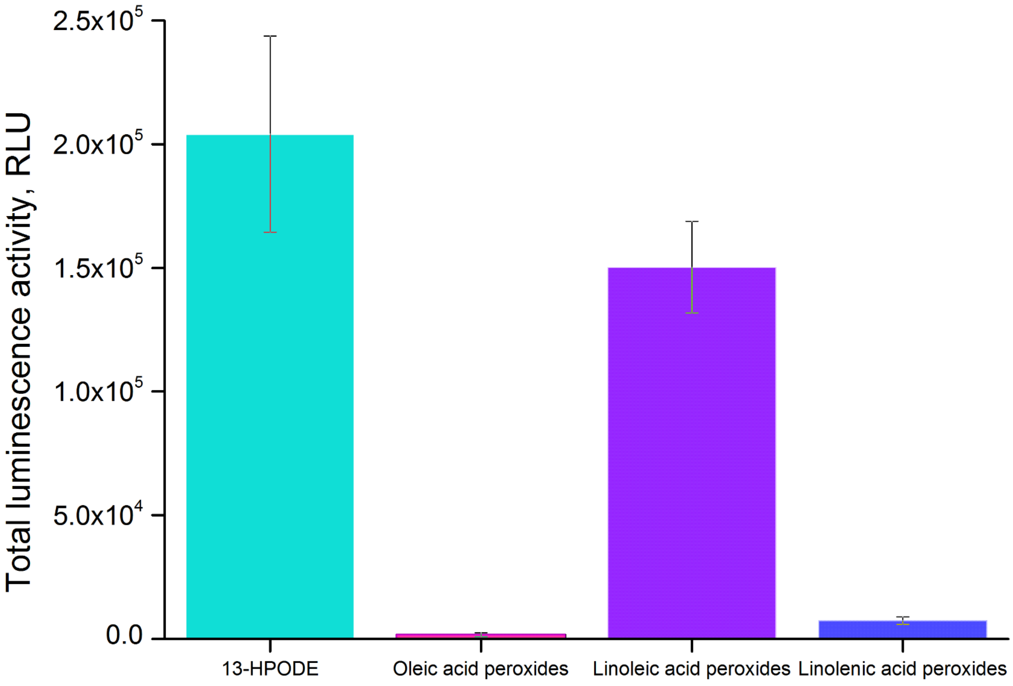

Figure S14. Bioluminescent activity of mixtures of peroxides of oleic, linoleic and linolenic acids obtained by oxidation with singlet oxygen in comparison with 13-HPODE. All experiments were performed in triplicate. The data are expressed as mean ± SD (n=3) and analyzed using Student's t-test. Differences were considered significant at $P < 0.05$.

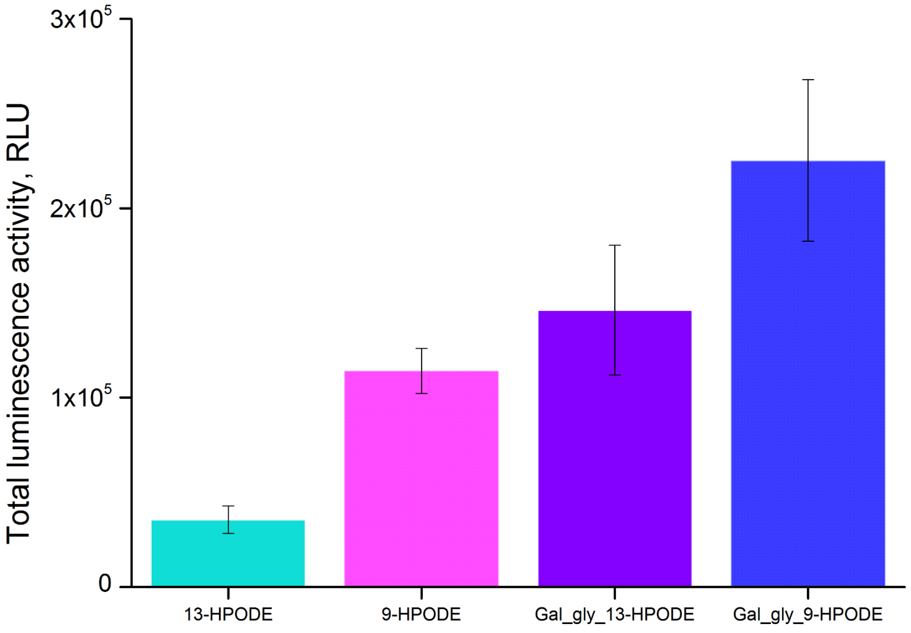

Figure S15. Bioluminescent activity of 9- and 13-HPODE, Gal_gly_9-HPODE and Gal_gly_13-HPODE. All experiments were performed in triplicate. The data are expressed as mean ± SD (n=3) and analyzed using Student's t-test. Differences were considered significant at $P < 0.05$.

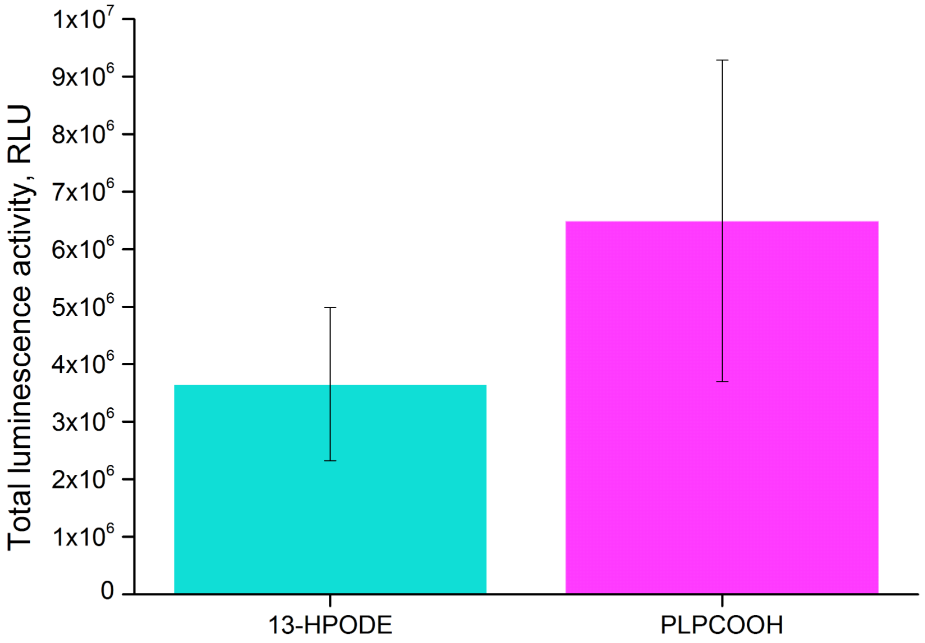

Figure S16. Bioluminescent activity of 1-palmitoyl-2-linoleoyl-sn-glycero-3-phosphocholine hydroperoxide (PLPCOOH) in comparison with 13-HPODE. All experiments were performed in triplicate. The data are expressed as mean ± SD (n=3) and analyzed using Student's t-test. Differences were considered significant at $P < 0.05$.
